# Intensification of Antiretroviral Therapy with a CCR5 Antagonist in Patients with Chronic HIV-1 Infection: Effect on T Cells Latently Infected

**DOI:** 10.1371/journal.pone.0027864

**Published:** 2011-12-08

**Authors:** Carolina Gutiérrez, Laura Díaz, Alejandro Vallejo, Beatriz Hernández-Novoa, María Abad, Nadia Madrid, Viktor Dahl, Rafael Rubio, Ana M. Moreno, Fernando Dronda, José Luis Casado, Enrique Navas, María Jesús Pérez-Elías, Javier Zamora, Sarah Palmer, Eduardo Muñoz, María Ángeles Muñoz-Fernández, Santiago Moreno

**Affiliations:** 1 Infectious Diseases Department, Hospital Universitario Ramón y Cajal, and IRYCIS, Madrid, Spain; 2 Inmunobiology Laboratory, Hospital General Universitario Gregorio Marañón, Madrid, Spain; 3 Karolinska Institute, Stockholm, Sweden; 4 Infectious Diseases Unit, Hospital General Universitario Doce de Octubre, Madrid, Spain; 5 Biostatistics Department, Hospital Universitario Ramón y Cajal, and IRYCIS, Madrid, Spain; 6 Immunology Department, Universidad de Córdoba, Córdoba, Spain; University of Toronto, Canada

## Abstract

**Objective:**

The primary objective was to assess the effect of MVC intensification on latently infected CD4^+^ T cells in chronically HIV-1-infected patients receiving antiretroviral therapy.

**Methods:**

We performed an open-label pilot phase II clinical trial involving chronically HIV-1-infected patients receiving stable antiretroviral therapy whose regimen was intensified with 48 weeks of maraviroc therapy**.** We analyzed the latent reservoir, the residual viremia and episomal 2LTR DNA to examine the relationship between these measures and the HIV-1 latent reservoir, immune activation, lymphocyte subsets (including effector and central memory T cells), and markers associated with bacterial translocation.

**Results:**

Overall a non significant reduction in the size of the latent reservoir was found (p = 0.068). A mean reduction of 1.82 IUPM was observed in 4 patients with detectable latent reservoir at baseline after 48 weeks of intensification. No effect on plasma residual viremia was observed. Unexpectedly, all the patients had detectable 2LTR DNA circles at week 24, while none of them showed those circles at the end of the study. No changes were detected in CD4^+^ or CD8^+^ counts, although a significant decrease was found in the proportion of HLA-DR^+^/CD38^+^ CD4^+^ and CD8^+^ T-cells. LPS and sCD14 levels increased.

**Conclusions:**

Intensification with MVC was associated with a trend to a decrease in the size of the latent HIV-1 reservoir in memory T cells. No impact on residual viremia was detected. Additional studies with larger samples are needed to confirm the results.

**Trial Registration:**

ClinicalTrials.gov NCT00795444

## Introduction

Antiretroviral therapy (ART) can reduce plasma HIV-1 RNA levels to <50 copies/ml [1]. However, low residual viremia, which is only detectable using ultrasensitive assays, can persist despite ART [2]–[4]. The origin and clinical implications of persistent low-level viremia are uncertain. While some studies postulate that it may be the result of virus released from latently infected cells [5]–[7], others support that it could arise from ongoing viral replication, as a consequence of incomplete inhibitory activity or penetration of antiretroviral drugs [8]–[13].

Although intensification of current ART with potent drugs could potentially decrease residual viremia and prevent replenishment of viral reservoirs, prior intensification studies have not demonstrated any impact on residual viremia [12], [14]–[17]. Only one study confirmed a transient increase in episomal 2LTR DNA circles after raltegravir intensification [18] as an indicator of recent HIV-1 replication [19]–[21], and another study showed a decrease in the frequency of episodes of intermittent viremia [22]. To our knowlegede, only a few studies have previously evaluated the effect of intensification of stable therapy on the latent reservoir of resting CD4^+^ T cells in chronically HIV-1-infected patients with controversial results. Ramratnam et al. found an accelerated decay of the HIV-1 latent reservoir after intensification therapy with abacavir with or without efavirenz [22]. Two more studies that measured the impact on the CD4^+^ T cell reservoir in patients receiving a four-drug combination as initial antiretroviral therapy provided discordant results, namely, a reduction in the reservoir in patients treated during acute infection [23] and no changes in chronically infected patients [24].

Bacterial translocation and a low level of ongoing viral replication have been associated with an increase in immune activation in HIV-1-infected patients [25], [26]. Evidence of increased bacterial translocation from damaged intestinal tissue was recently demonstrated in chronic HIV-1 infection [25], [27]. In acute HIV-1 infection, intestinal CD4^+^ T cells are rapidly depleted and T-cell activation is high. Since monitoring intestinal cells is difficult, expression of surrogate markers such as the mucosal homing receptor α4β7 integrin on circulating T cells has been proposed to correlate with loss or restoration of intestinal CD4^+^ T cells and could prove helpful in monitoring the success of therapeutic strategies [28]–[30]. Besides, recent studies have shown that HIV-1 utilizes α4β7 integrin to bind to CD4^+^ T cells [31].

Maraviroc (MVC) is a potent new antiretroviral agent approved for the treatment of HIV-1 infection that blocks interaction between the virus and the CCR5 co-receptor, a crucial step in the HIV-1 life cycle [32]. Previous clinical trials have demonstrated the safety, tolerability, and efficacy of MVC in both treatment-naive and treatment-experienced patients [33], [34].

We performed a prospective open-label pilot phase II clinical trial to assess the effect of MVC intensification on latently infected CD4^+^ T cells in chronically HIV-1-infected patients receiving antiretroviral therapy. We also analyzed residual viremia and episomal 2LTR DNA to examine the relationship between these measures and the HIV-1 latent reservoir, immune activation, lymphocyte subsets (including effector and central memory T cells), and markers associated with bacterial translocation. The hypothesis was that if these factors are causally linked, altering one of them with the intervention should result in alteration of the others.

## Methods

The protocol for this trial and supporting CONSORT checklist are available as supporting information; see Checklist S1 and Protocol S1.

### Study Design

We performed an open-label pilot phase II clinical trial to evaluate the effect of MVC on the cellular HIV-1 reservoir in patients receiving ART. The study was conducted at Hospital Universitario Ramón y Cajal in Madrid, Spain between 2008 and 2010. This independent clinical trial (NCT00795444) had a follow up of 48 weeks of intensification with MVC (developed and provided by Pfizer, Inc.).

### Ethics Statement

The study was carried out according to the recommendations of the Declaration of Helsinki and current Spanish legislation on clinical trials. It was approved by the AEMPS (Spanish Agency for Medications and Health Products) and our Independent Ethics Committee (Hospital Ramón y Cajal, 28034 Madrid, Spain; ceic.hrc@salud.madrid.org). All patients provided their written informed consent for participation, which included sample collection and laboratory determinations.

### Patients and specimen collection

Nine patients met all the following inclusion criteria: undetectable plasma viral load (pVL) by standard commercial assays (<50 copies HIV-1 RNA/ml) for at least two years; ART with three or more drugs for at least two years; CD4^+^ T lymphocyte count > 350 cells/mm^3^; R5 viral tropism testing a pretreatment sample using a phenotypic assay (Trofile®, Monogram Biosciences, C.A), and no previous treatment with MVC. Patients were recruited from two hospitals (Hospital Ramón y Cajal and Hospital Doce de Octubre), both in Madrid, Spain.

**Figure 1:**
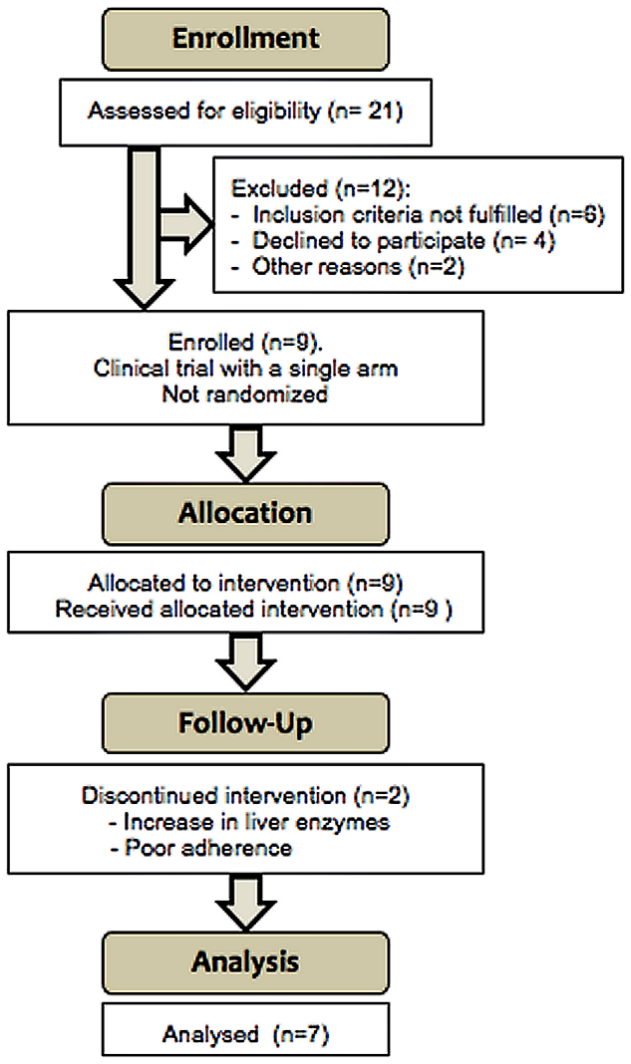
Consort Flow Diagram.

Patient's enrollment, allocation and follow up are described in figure 1.

Samples were collected at baseline and after 12, 24, 36, and 48 weeks of intensification with MVC. A total of 300 ml of heparinized whole blood was drawn to quantify the latent HIV-1 reservoir; 50 ml of whole blood with EDTA was drawn to obtain plasma and isolate peripheral blood mononuclear cells (PBMC). Samples were cryopreserved in the HIV Biobank of the Spanish AIDS Research Network (RIS) following current procedures [35].

In addition, two control groups (10 treatment-naive HIV-1-infected patients, and 10 HIV-1-negative subjects) were also analyzed for some measurements.

### Latently HIV-1-infected memory T cells

Two baseline determinations (three months apart) were performed to investigate the accuracy of the method and the stability of the reservoir.

We carried out a previously described co-culture assay [36], [37]. Briefly, PBMC were isolated from 300 ml of heparinized whole blood using Ficoll density gradient centrifugation (Lymphocytes Isolation Solution, Rafer, S.L Zaragoza, Spain). Resting CD4^+^ T cells (CD4^+^/HLA-DR^-^/CD25^-^) were isolated and purified using magnetic beads according to the manufacturer's recommendations (Miltenyi Biotec, S.L. Bergisch Gladbach, Germany). CD4^+^ T-cell purity greater than 99% with less than 0.4% activation was confirmed by flow cytometry. The isolated resting CD4^+^ T cells were plated in replicate limiting dilutions and co-cultured with allogenic irradiated PBMC from seronegative donors and phytohemagglutinin. The activated cell culture was fed once a week with PBMC from healthy donors after depletion of CD8^+^ T cells. On days 15 and 21, culture supernatants were tested for HIV-1 replication using the HIV-1 p24 antigen assay (Innogenetics Diagnostica Iberia, S.L Tarragona, Barcelona, Spain). Infected cell frequencies were determined by the maximum likelihood method and expressed as infectious units per million (IUPM) resting CD4^+^ T cells [38]. The millions of purified resting CD4^+^ T cells obtained from the total PBMCs conditioned the number of replicates of each dilution. The assay was performed to each point as duplicate fivefold dilution series.

From baseline to week 12 of follow-up, the limit of detection of the assay was 0.12 IUPM resting CD4^+^ T cells. Thereafter, the limit of detection decreased to 0.023 IUPM, since the total blood extraction volume was increased after approval of the amended protocol by the IEC.

### Residual viremia

Residual viremia was measured with internally controlled an ultrasensitive quantitative real-time RT-PCR (single copy assay), as reported elsewhere [39]. The optimal HIV-1 RNA detection threshold was 0.3 copies/ml when 5 to 7 ml of starting plasma was available. We used a median of 6.5 ml (IQR: 4.6 to 7).

### HIV-1 episomal 2LTR DNA circles

The presence of HIV-1 episomal 2LTR DNA circles was detected by qualitative PCR. To maximize the recovery of 2LTR DNA circles and overcome the lack of sensitivity of this technique [8], [18], [40] enriched 2LTR episomal DNA circles were extracted selectively from ∼5 million PBMC using QIAprep Spin Miniprep (Qiagen, Valencia, California, USA) following the manufacturer's protocol for low-copy-number plasmids, as previously described [8].

A nested PCR flanking the episomal 2LTR DNA circle junction was designed. In the first round, 5–20 µL of episomal DNA were amplified in a 50-µL reaction with the following primers: forward, 5′-TAAGATGGGTGGCAAGTGGTCA; and reverse, 5′-TCTACTTGTCCATGCATGGCTT.

The second PCR round was performed using 1 to 2 µL of the first reaction product as the template and primers spanning the unique junction formed by ligation of 5′ and 3′ LTR sequences (forward, 5′-AATCTCTAGCAGTACTGGAAG; reverse, 5′-GCGCTTCAGCAAGCCGAGTCCT). PCR products were analyzed on a 1% agarose gel stained with GelRed (Biotium, Hayward, California, USA).

### Immune activation and lymphocyte subsets

Fresh EDTA anticoagulated whole blood was used to analyze CD4^+^ and CD8^+^ T cell activation with the following antibody combination: CD3-allophycocyanin (APC)-Cy7, CD4-peridinin chlorophyll protein complex (PerCP), CD8-phycoerythrin (PE)-Cy7, CD38-phycoerythrin (PE), and HLA-DR-allophycocyanin (APC). Lymphocyte subpopulations were defined as naïve (CD45RA+CCR7+), T central memory (CD45RA-CCR7+, TCM), T effector memory (CD45RA-CCR7-, TEM), and T effector memory RA+ (CD45RA+CCR7-, TemRA). To analyze lymphocyte subsets this antibody combination was used: CD3-allophycocyanin (APC)-Cy7, CD4-peridinin chlorophyll protein complex (PerCP), CD8-phycoerythrin (PE)-Cy7, CD45RA- phycoerythrin (PE), and CCR7-allophycocyanin (APC), β7-allophycocyanin (APC). Antibodies were from Becton Dickinson (Becton Dickinson, NJ, USA), and an unstained control was performed for all samples. Briefly, 100 µL of blood were lysed with 200 µl of FACS Lysing solution (Becton Dickinson) for 30 min at room temperature, incubated with the antibodies during 20 min at 4°C, washed and resuspended in PBS containing 1% azida. Cells were analyzed in a Gallios flow cytometer (Beckman-Coulter, CA, USA). At least 15000 CD3^+^ cells were collected for each sample and analyzed with Kaluza software (Beckman-Coulter) initially gating lymphocytes according to morphological parameters. The gating was always the same between different time points.

### Bacterial translocation

Two commercial assays were used to evaluate bacterial translocation from plasma samples. Plasma bacterial lipopolysaccharide (LPS) was measured using QCL-1000 Limulus Amebocyte Lysate (Lonza®, Basel, Switzerland) according to the manufacturer's protocol. This test quantifies endotoxins from the cell wall of gram-negative bacteria. Plasma sCD14 was quantified using the Quantikine® Human sCD14 Immunoassay (R&D Systems, Minneapolis, Minnesota, USA), according to the manufacturer's instructions. Samples were run in duplicate in all cases.

### Statistical analysis

This is an exploratory pilot study that is trying to prove a concept. A treatment group of 10 patients has been considered adequate, based on data from previous studies published by other authors on the same subject.

Continuous variables were expressed as the median and interquartile range and discrete variables as percentages. The *t* test for independent samples was used to compare normally distributed continuous variables and the Mann-Whitney test to compare non-normally distributed continuous variables. Categorical variables were described as proportions. The association between categorical variables was evaluated using the chi-square test. A Spearman correlation was used. Statistical analysis was performed using SPSS software 16.0 (Inc., Chicago, Illinois, USA).

## Results

### Patient characteristics

The baseline characteristics of the patients are summarized in table 1. The median age was 46 years and 8 patients were male. The median baseline CD4^+^ and CD8^+^ T-cell counts were 711 and 784 cells/mm^3^, respectively. All patients were receiving nucleoside reverse transcriptase inhibitor (NRTI)–containing regimens combined with non-nucleoside reverse transcriptase inhibitors (NNRTIs) in two cases (22%), with protease inhibitors (PIs) in five cases (56%), and with a third nucleoside in two cases (22%). The median duration of ART before study entry was 75 months.

**Table 1:** Baseline characteristics of the patients.

Patient ID	Age (years)	Gender	Risk Factors	Viral load (copies/ml)	CD4 count (cells/mm^3^)	CD8 count (cells/mm^3^)	Duration of ART (months)	Current ART regimen	MVC intensification (mg/BID)
MVC 1	49	M	IDU	<50	1169	683	124	ddI+3TC+NVP	300
MVC 2	47	M	MSM	<50	486	673	75	FTC+TDF+ATV/r	150
MVC 3	46	M	IDU	<50	796	650	78	3TC+ABC+ATV	150
MVC 4	46	F	HSx	<50	534	784	57	AZT+3TC+ABC	300
MVC 5	45	M	IDU	<50	728	639	142	AZT+3TC+ABC	300
MVC 6	58	M	MSM	<50	787	1109	144	FTC+TDF+LOP/r	150
MVC 7	32	M	MSM	<50	694	1608	50	FTC+TDF+ATV/r	150
MVC 8	30	M	MSM	<50	589	1064	42	FTC+TDF+EFV	600
MVC 9	30	M	MSM	<50	1241	1414	38	TC+TDF+LOP/r	150
**Median [IQR]**	46 [31]–[48]	-	-	<50	711 [547–793]	784 [673–1109]	75 [38–144]	-	-

ddI: didanosine; NVP: nevirapine; FTC: emtricitabine; TDF: tenofovir; ATV/r: atazanavir/boosted with ritonavir; ABC: abacavir; AZT: zidovudine; LOP/r: lopinavir/boosted with ritonavir; EFV: efavirenz; F: Female; M: Male; HSx, Heterosexual; MSM: men who have sex with men.

MVC was well tolerated. Two patients interrupted the study early. Data from these patients were included only until the study interruption. None of them was included in the final analysis at week 48.One patient, who was coinfected with hepatitis C virus, had a significant increase in liver enzymes after the visit at week 12; the other discontinued all antiretroviral drugs due to poor adherence after the visit at week 12. The remaining patients maintained an HIV viral load <50 copies/ml until the end of the study.

### Effect of MVC intensification on latent HIV-1 reservoir

**Figure 2:**
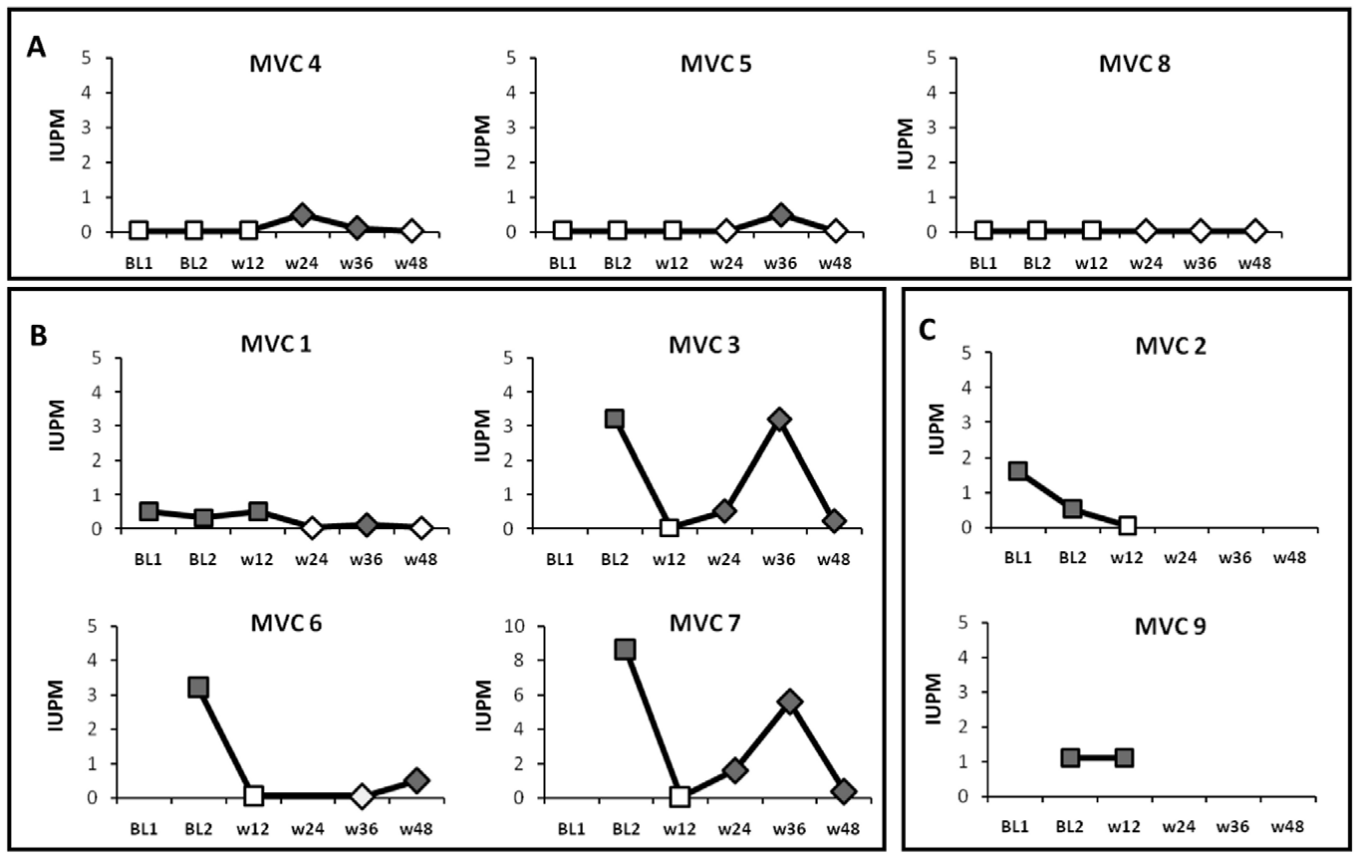
Effect of MVC intensification on HIV-1 latently infected memory CD4^+^ T cells. (**a**) Patients who had a latent reservoir below the limit of detection at baseline, and remained undetectable after 48 weeks of intensification. (**b**) Patients with quantifiable latent reservoir at baseline, and decreased after 48 weeks of intensification, (**c**) patients with quantifiable latent reservoir at baseline but discontinuated the study after w12. Square symbols: detection limit of 0.12 IUPM. Diamond symbols: detection limit of 0.023 IUPM. Open symbols: under the limit of detection.

The number of latently HIV-1-infected memory CD4^+^ T cells decreased after 48 weeks of MVC intensification, although the difference was not significant (p = 0.068) (figure 2). Three patients showed a reservoir below the limit of detection before receiving MVC (figure 2A), and this remained undetectable after 48 weeks despite a transient increase in two patients. The remaining patients, all of whom had a quantifiable reservoir at study entry, showed a decrease in the IUPM after 48 weeks of intensification (figure 2B) or after 12 weeks of intensification (figure 2C, two patients who discontinued the study after week 12). A mean reduction of 1.82 IUPM was found in the 4 patients with detectable latent reservoir at baseline after 48 weeks of intensification with MVC.

### Effect of MVC intensification on residual viremia

The impact on the cellular reservoir was not associated with significant changes in residual viremia. At baseline, 8 of the 9 patients had undetectable residual viremia using the single copy assay. A significant increase in residual viremia was observed after 12 weeks of MVC intensification compared to baseline (p = 0.027). Afterwards, residual viremia decreased progressively until the end of the study, with no significant differences compared to baseline (p = 0.08, table 2).

**Table 2:** Effect of MVC intensification on residual viremia and episomal DNA circles.

Patient	SCA					Episomal 2LTR DNA				
ID	Baseline	w12	w24	w36	w48	Baseline	w12	w24	w36	w48
MVC 1	0.3	S.F.	S.F.	<0.3	<0.3	-	+	+	-	-
MVC 2	<0.3	<0.3	N.D.	N.D.	N.D.	-	+	N.D.	N.D.	N.D.
MVC 3	<0.3	SF	<0.5		0.5	-	-	+	-	-
MVC 4	<0.3	9.2	<0.4	<0.3	<0.3	-	-	+	-	-
MVC 5	<0.3	1.0	<0.5	<0.3	2.2	-	-	+	-	-
MVC 6	<0.3	3.0	3.0	1.1	3.3	-	+	+	-	-
MVC 7	<0.6	7.0	<0.5	<0.5	<0.5	-	-	+	+	-
MVC 8	<0.4	0.5	4.6	<0.5	3.7	-	+	+	-	-
MVC 9	<2.0	1.0	N.D.	N.D.	N.D.	-	+	N.D.	N.D.	N.D.
Median	0[0–0]	1[0.5–7]	0[0–3.4]	0[0–0.2]	0.5[0–3.3]					
p *		0.027	0.1	0.6	0.08		0.037	0.012		

SCA: Single copy assay; S.F.: Standard failure; N.D.: Not determined; *: Statistical significance compared to baseline (significance p<0.05).

### Effect of MVC intensification on episomal 2LTR DNA circles

At baseline, episomal 2LTRs DNA circles were undetectable in all patients. However, at week 12 they were detected in 5 patients (p = 0.037, compared to baseline) and at week 24 in all 9 patients (p = 0.012, compared to baseline). At week 48, episomal 2LTR DNA circles were again undetectable in all patients (table 2).

### Effect of MVC intensification on T-cell activation

**Figure 3:**
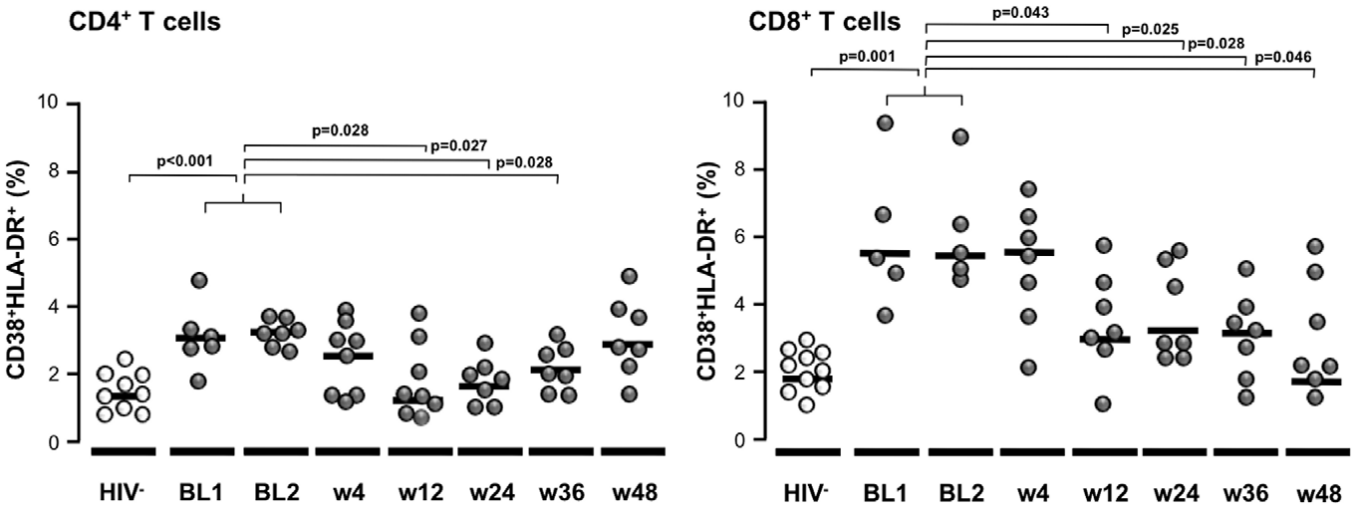
Immune activation during MVC intensification. CD4^+^ and CD8^+^ T cell activation of the patients during the follow up is shown. A group of HIV-1 negative individuals are also shown.

The level of activation of CD4^+^ T cells decreased significantly after 12, 24, and 36 weeks of MVC intensification (p = 0.028, p = 0.027, and p = 0.028, respectively), only to increase after 48 weeks, although the difference with baseline was not significant (p = 0.6) (figure 3). Compared to HIV-negative subjects, CD4^+^ T-cell activation at baseline was significantly higher (p<0.001), with no significant differences at weeks 12 and 24 (p = 0.950 and p = 0.181, respectively).

The level of activation of CD8^+^ T cells also decreased significantly after 12 weeks and until the end of the study (p = 0.043, p = 0.025, p = 0.028, and p = 0.046, at weeks 12, 24, 36, and 48, respectively) (figure 3). The level of cell activation at baseline was higher than in the HIV-negative subjects (p = 0.001). Only at week 48 was this difference not significant compared to the HIV-negative group (p = 0.181).

### Effect of MVC intensification on T-cell subsets

**Figure 4:**
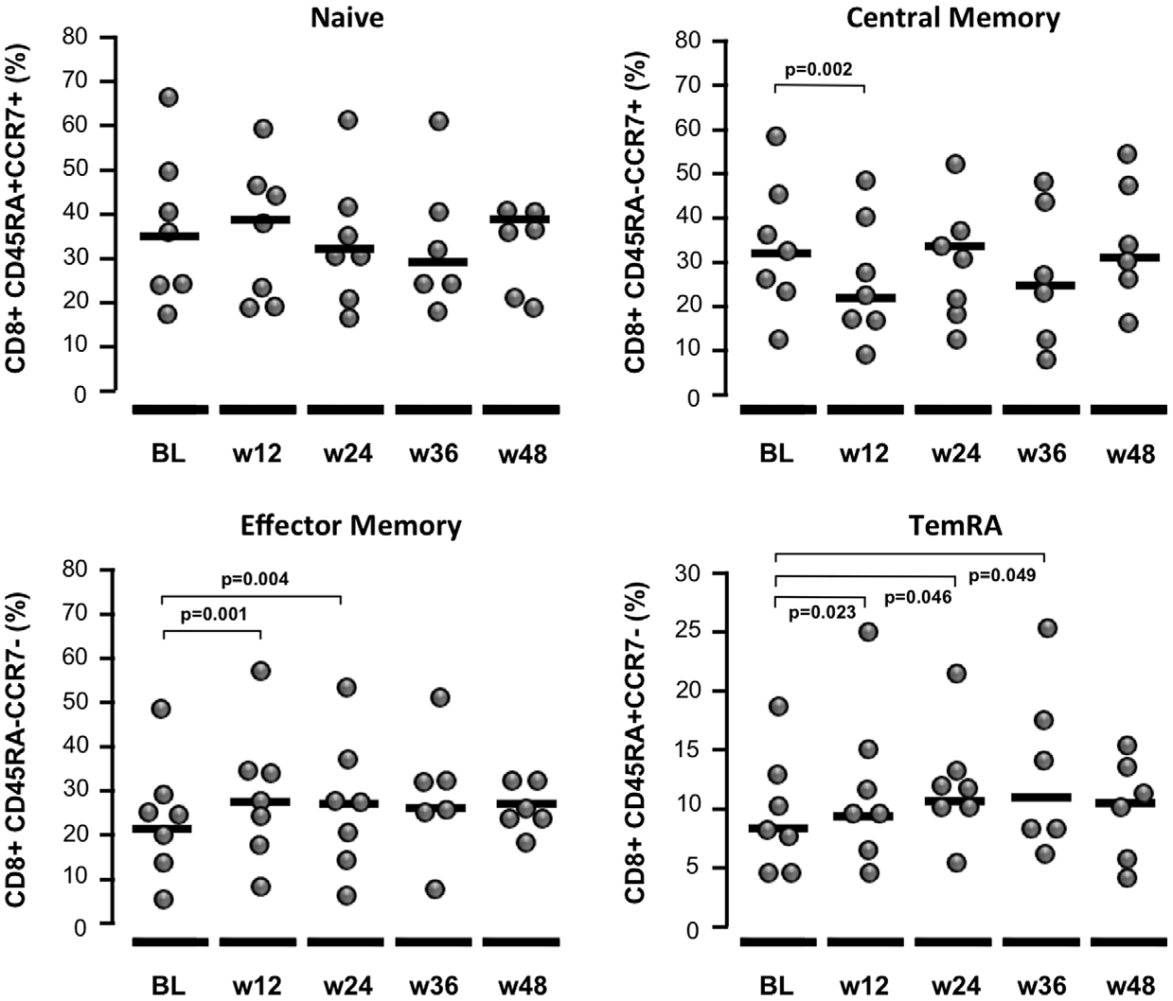
CD8 ^+^ **T cell subpopulations after MVC intensification.** The proportion of naïve, central memory (TCM), effector memory (TEM), and effector memory RA^+^ (TemRA) cells is shown.

No differences in the absolute number or percentage of CD4^+^ or CD8^+^ T cells were found during follow-up. A significant increase in CD8^+^ effector memory and TemRA T cell count was found during the first 24 and 36 weeks after MVC intensification, respectively. Thereafter, the differences compared to baseline were not significant (figure 4). On the other hand, a significant decrease in CD8^+^ central memory T cells was observed at week 12 (p = 0.002). The level of CD8^+^ naive T cells was similar during follow-up (p>0.05 at all time points). No significant differences were observed in any CD4^+^ T cell subsets during follow-up (data not shown).

**Figure 5:**
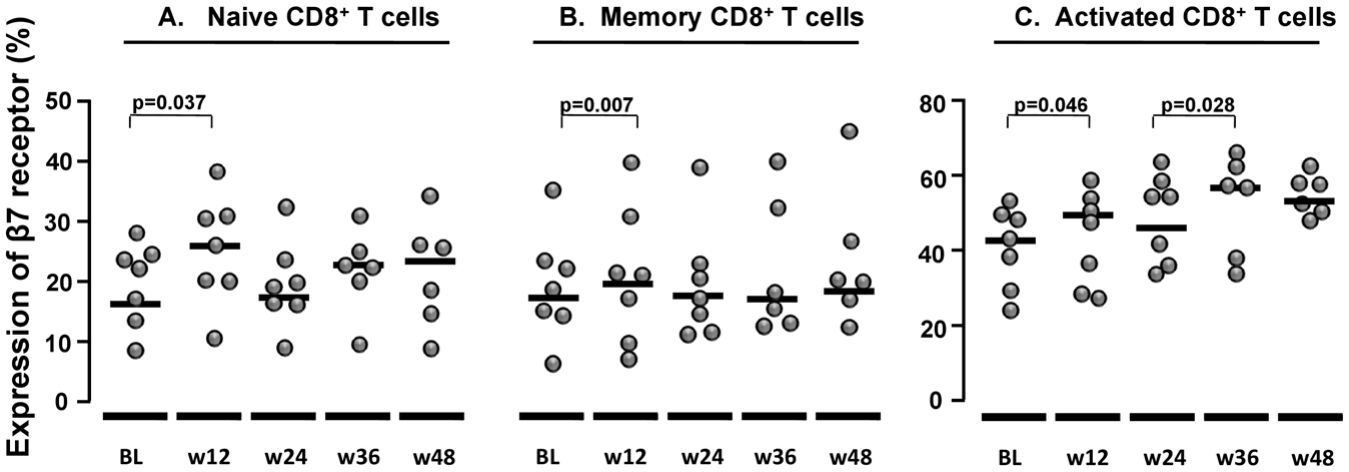
Proportion of CD8 ^+^ **T cells expressing** β**7 receptor.** A) naïve T cells, B) memory T cells, and C) activated T cells.

The proportion of CD8^+^ naïve and memory T cells expressing a β7 receptor significantly increased after 12 weeks of intensification (p = 0.037 and p = 0.007, respectively) (figure 5A and 5B respectively). The expression of β7 receptor also increased in activated CD8^+^ T cells at week 12 compared to baseline (p = 0.046), and a trend was observed during the rest of the follow up (figure 5C).

### Effect of MVC intensification on bacterial translocation

**Figure 6:**
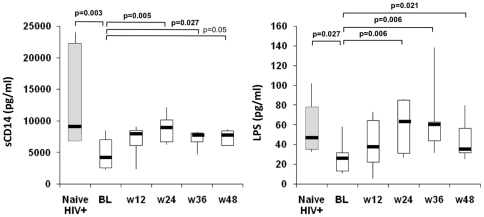
Bacterial translocation. Levels of sCD14 and LPS during the follow up are shown. The levels of a group of HIV-1 positive naïve for ART are also included.

A significant increase in sCD14 level was observed after 24, 36 and 48 weeks of intensification with MVC compared to baseline (p = 0.015, p = 0.028 and p = 0.028, respectively) (figure 6). These levels were significantly lower than those of naive HIV-1-infected patients at all time points. LPS levels were also higher at weeks 24, 36, and 48 of intensification than at baseline (p = 0.006, p = 0.006, and p = 0.021, respectively) (figure 6). The level of LPS at baseline was significantly lower that that of treatment-naive HIV-1-infected patients; during follow-up, these levels were no different from those of the treatment-naive patients.

A significant direct correlation was found between sCD14 levels and LPS levels at baseline (p = 0.035, r = 0.743) while a similar trend was observed during the rest of the follow up. Furthermore, the LPS level correlated directly with both the proportion of CD4^+^ T cells expressing a β7 receptor and the level of naive CD4^+^ T cells expressing β7 receptor at week 24 (p = 0.047, r = 0.817). Similarly, the level of LPS at week 36 correlated directly with that of CD8^+^ effector memory T cells (p = 0.045, r = 0.818).

## Discussion

In this pilot study, intensification of treatment with MVC in patients chronically infected with HIV-1 receiving successful long-term ART was associated with a trend to an accelerated decay in the pool of latently infected CD4^+^ T cells. No impact on residual viremia was observed.

Intensification of successful antiretroviral therapy has been explored in two different ways. Some studies have evaluated its role for clinical purposes, that is, to achieve better virological control or a greater increase in CD4^+^ T-cell counts, or, more recently, to reduce immune activation. With the exception of one study that showed a decrease in HIV RNA levels after abacavir intensification, none of the studies has been able to show significant improvements with intensified therapy compared to the standard three-drug regimens [11], [12], [14]–[17], [41]. As a consequence, most clinicians feel that no further research in this direction is necessary. Other studies have evaluated intensification as a strategy to help to eradicate HIV. The rationale for this approach is that suppression of putative replication could reduce residual viremia and replenishment of cellular reservoirs leading to a decrease in the total latent reservoir [8]–[18], [41]. The results of all the studies are uniform in that no single group has shown a significant reduction in plasma HIV RNA as a result of intensification. This finding could be interpreted as confirmation of the lack of residual viral replication, thus supporting the hypothesis that residual viremia is the result of viral release from stable reservoirs and not the consequence of new rounds of viral replication [5]–[7]. An alternative hypothesis would be that the effect of intensification takes place mainly outside plasma sites and, therefore, the measurement of plasma viral load only could not detect this effect. Some of the studies that included measurements other than plasma HIV RNA seem to support this hypothesis [42]–[44].

We observed a reduction in the size of the latent reservoir after intensification of treatment with MVC, although this reduction was not significant, probably due to the small sample size. This finding is consistent with other studies that included patients with different baseline characteristics [22], [23], although some authors do not support it [24]. The decline in the latent reservoir that we found does not appear to be secondary to a decrease in residual viral replication in plasma, since there were no detectable changes in the measurements of residual viremia. It has been suggested that a dilutional effect could account for the reduction observed, but the lack of an increase in the absolute count and the percentage of both CD8^+^ and CD4^+^ T cells strongly argues against this possibility.

Other findings in our study may help explain the effect on the latent reservoir. Unexpectedly, 2LTR DNA circles were transiently detectable during MVC intensification. Due to its mechanism of action, MVC is not expected to increase 2LTR DNA circles, even when inhibiting persistent viral replication. The increase in levels of this marker suggests that treatment with MVC in patients with undetectable viral load may induce viral replication. Preclinical studies showed that MVC had no agonistic activity on activated CD4^+^ T cells and, therefore, could not induce viral replication [32]. Later reports, however, have shown that the possibility of acting as a partial agonist on CCR5 could be dependent on cell type [45]. Therefore, MVC could activate CCR5 in resting CD4^+^ T cells and, through intracellular signalling, increase transcriptional activation of the latent virus. As an alternative hypothesis, CCR5 blockade by MVC could lead natural ligands or chemokines to bind to other receptors and induce latent HIV transcriptional activation through distinct signalling pathways. Both hypotheses would be consistent with the reduced number of latently infected memory T cells found in this study, the transient increase in the number of copies of HIV RNA seen in some patients, and the increased bacterial translocation plus migration of α4β7 cells to the gut. This possibility is further supported by the increased immune activation in intestinal tissue biopsies that was observed in another MVC intensification study [46]. Finally, trafficking of cells to peripheral blood from some other compartment cannot be discarded to explain some of the results, including the increase in 2 LTR circles.

Neither of the above hypotheses can account for the diminished immune activation that we detected. MVC has been shown to decrease immune activation in clinical trials, and it could be assumed that a different mechanism drives the impact on immune activation [47]. The decrease in immune activation does not correlate with a decrease in bacterial translocation, which in fact increased during the study, thus supporting increased viral replication and activation in the intestine resulting from the release of proinflammatory cytokines [48].

The transient increase in TEM and TemRA CD8^+^ T cell counts parallels the increase in bacterial translocation. These increments are balanced by a transient decrease in central memory CD8^+^ T cell counts, while naive CD8^+^ T cell levels remain stable [49]. In addition, the finding of increased β7 receptor levels on CD8^+^ T cells and especially on activated CD8 T cells support the idea of migration of these cells to the intestine, where bacterial translocation takes place. This migration of activated cells to the gut could account for the reduced immune activation observed in the periphery. In this sense, immune events detected in peripheral blood may not reflect what takes place in the intestine, as is the case for the huge CD4^+^ T-cell destruction that occurs in the intestine of the SIV rhesus macaque during acute infection, which is not reflected in lymph nodes or peripheral blood [50].

Our work has a series of limitations. First, it is a pilot study with a small sample size, thus limiting the strength of the conclusions. Second, no control group was included. The study was designed as a proof of concept with no guarantee of any spontaneous effect in the sample. Finally, the methodological tools used for the different measurements and their interpretations are still imperfect.

In summary, our study shows positive effects of MVC intensification on latently HIV-1-infected CD4^+^ T cells. These effects are not reflected in reduced residual plasma viremia and may be associated with the activity of the drug in an anatomical reservoir, most likely the intestine. The mechanism by which MVC decrease the HIV-1 reservoir is under investigation. The decrease we observed, however, is by no means large enough to eradicate HIV. New therapeutic strategies, probably based on anti-latency drugs, are necessary to eradicate viral reservoirs. In this sense, complete inhibition of viral replication at all sites can be presumed to be necessary before these new treatment strategies can be applied. The exact role of treatment intensification in eradication remains undefined, and a large, comparative trial with adequate methodology is needed to provide a definitive answer to this relevant question.

## Supporting Information

Protocol S1 **Trial Protocol.**
(DOC)

Checklist S1 **CONSORT Checklist.**
(DOC)
