# Supplementary material for: Intensification of Antiretroviral Therapy with a CCR5 Antagonist in Patients with Chronic HIV-1 Infection: Effect on T Cells Latently Infected
Source: PLoS One. 2011 Dec 8;6(12):e27864. doi: 10.1371/journal.pone.0027864 (PMC3234247; doi:10.1371/journal.pone.0027864)
Supplement: Protocol S1 — Trial Protocol. (DOC) [file pone.0027864.s001.doc]

| 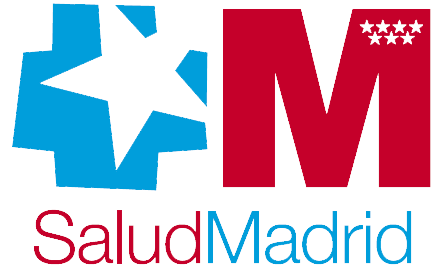 | **Hospital Universitario**  **Ramón y Cajal**  **Servicio de Enfermedades Infecciosas**  s | 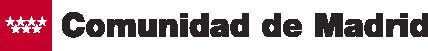 |
| --- | --- | --- |

**PROTOCOL**

**ERRADVIH-01**

Versión 5.0 (31 Enero 2010)

Eudra CT 2007-003995-21

# PILOT STUDY OF THE EFFECT OF A CCR5 CORECEPTOR ANTAGONIST ON THE LATENCY AND RESERVOIR OF HIV-1 IN PATIENTS TAKING HIGHLY ACTIVE ANTIRETROVIRAL THERAPY

# INVESTIGATOR DECLARATION

- I accept the responsibility for ensuring that this trial is carried out correctly at the study center.
- I agree to carry out the study according to this protocol.
- I will not make any changes to the protocol without the previous consent of the sponsor and the authorization of the Institutional Review Board, except when it is necessary to counter any imminent danger to a patient or as far as the administrative aspects of the trial are concerned (and when permitted by current law to this effect).
- I know and will fulfill the norms of Good Clinical Practice (GCP) and all other relevant legal requirements

Signed: Date:

Chief Investigator

# TABLE OF CONTENTS

***INVESTIGATOR DECLARATION*** [***2***](#__RefHeading___Toc183666247)

TABLE OF CONTENTS [3](#__RefHeading___Toc183666248)

1. SYNOPSIS [6](#__RefHeading___Toc183666249)

2. GENERAL INFORMATION [8](#__RefHeading___Toc183666250)

**2.1.** **Identification of the trial** [8](#__RefHeading___Toc183666251)

**2.2.** **Type of clinical trial** [8](#__RefHeading___Toc183666252)

**2.3.** **Study products** [8](#__RefHeading___Toc183666253)

**2.4.** **Sponsor data** [8](#__RefHeading___Toc183666254)

**2.5.** **Technical director responsible for preparing and controling the samples** [8](#__RefHeading___Toc183666255)

**2.6.** **Monitor** [8](#__RefHeading___Toc183666256)

**2.7.** **Chief investigator and collaborators** [8](#__RefHeading___Toc183666257)

**2.8.** **Study center** [9](#__RefHeading___Toc183666258)

**2.9.** **Expected duration of the trial** [9](#__RefHeading___Toc183666259)

3. RATIONALE AND OBJECTIVES [10](#__RefHeading___Toc183666260)

**3.1.** **Rationale** [**¡Error! Marcador no definido.**](#__RefHeading___Toc183666261)

**3.2.** **Primary objective** [14](#__RefHeading___Toc183666262)

**3.3.** **Secondary objectives** [14](#__RefHeading___Toc183666263)

4. TYPE OF TRIAL AND DESIGN [14](#__RefHeading___Toc183666264)

**4.1.** **Clinical trial phase** [14](#__RefHeading___Toc183666265)

**4.2.** **Randomization process** [14](#__RefHeading___Toc183666266)

**4.3.** **Type of control and design** [14](#__RefHeading___Toc183666267)

**4.4.** **Blinding techniques** [14](#__RefHeading___Toc183666268)

**4.5.** **Enrollment, washout, and follow-up** [14](#__RefHeading___Toc183666269)

5. SELECTION OF SUBJECTS [15](#__RefHeading___Toc183666270)

**5.1.** **Inclusion criteria** [15](#__RefHeading___Toc183666271)

**5.2.** **Exclusion criteria** [15](#__RefHeading___Toc183666272)

**5.3.** **Number of subjects planned and rationale** [15](#__RefHeading___Toc183666273)

**5.4.** **Analysis and withdrawal criteria** [15](#__RefHeading___Toc183666274)

**5.5.** **Approximate duration of the enrollment period** [15](#__RefHeading___Toc183666275)

6. DESCRIPTION OF TREATMENT [16](#__RefHeading___Toc183666276)

**6.1.** **Definition of treatment** [16](#__RefHeading___Toc183666277)

**6.2.** **Concomitant treatment** [16](#__RefHeading___Toc183666278)

7. STUDY DEVELOPMENT AND EVALUATION OF RESPONSE [16](#__RefHeading___Toc183666279)

**7.1.** **Evaluation criteria.** [16](#__RefHeading___Toc183666280)

**7.2.** **Study development** [16](#__RefHeading___Toc183666281)

**7.3.** **Description of the methods for evaluating the response** [17](#__RefHeading___Toc183666282)

7.3.1. Laboratory determinations [17](#__RefHeading___Toc183666283)

8. ADVERSE EVENTS [18](#__RefHeading___Toc183666284)

**8.1.** **Minimum required information** [18](#__RefHeading___Toc183666285)

**8.2.** **Imputability criteria** [18](#__RefHeading___Toc183666286)

**8.3.** **Procedures for the notification of adverse events** [19](#__RefHeading___Toc183666287)

**8.4.** **Procedures for notification of severe and unexpected adverse events.** [19](#__RefHeading___Toc183666288)

9. ETHICS [20](#__RefHeading___Toc183666289)

**9.1.** **General and specific norms for investigators** [20](#__RefHeading___Toc183666290)

**9.2.** **Informed consent** [20](#__RefHeading___Toc183666291)

**9.3.** **Security and confidentiality** [20](#__RefHeading___Toc183666292)

**9.4.** **Content of the trial budget** [20](#__RefHeading___Toc183666293)

**9.5.** **Insurance** [20](#__RefHeading___Toc183666294)

10. PRACTICAL CONSIDERATIONS [21](#__RefHeading___Toc183666295)

**10.1.** **Responsibilities of the trial participants** [21](#__RefHeading___Toc183666296)

**10.2.** **Deviations from the protocol** [21](#__RefHeading___Toc183666297)

**10.3.** **Filing of the documentation** [21](#__RefHeading___Toc183666298)

**10.4.** **Identification of the samples** [22](#__RefHeading___Toc183666299)

**10.5.** **Amendments to the protocol** [22](#__RefHeading___Toc183666300)

**10.6.** **Acceptance by the investigator** [22](#__RefHeading___Toc183666301)

**10.7.** **Conditions for publication** [22](#__RefHeading___Toc183666302)

11. STATISTICAL ANALYSIS [22](#__RefHeading___Toc183666303)

12. APPENDICES [24](#__RefHeading___Toc183666304)

A. Declaration of Helsinki [24](#__RefHeading___Toc183666305)

C. Commitment from Chief Investigator and Collaborators [28](#__RefHeading___Toc183666306)

D. Informed Consent Form [29](#__RefHeading___Toc183666307)

**ANNEXES**

Annex I: Case Report Form

Annex II: Insurance Policy

Annex III: Investigator Brochure

Annex IV: Report on the Analysis of Samples and Procedures to Be Used

# SYNOPSIS

# CLINICAL TRIAL

# Pilot study of the effect of a CCR5 coreceptor antagonist on the latency and reservoir of HIV-1 in patients taking highly active antiretroviral therapy

**0. Type of application**

First trial for a new indication.

**1. Sponsor**

Fundación para la Investigación Biomédica del Hospital Ramón y Cajal.

Address: Carretera de Colmenar Viejo Km 9, 100

28034 Madrid

**Contact**

Itziar de Pablo López de Abechuco

Clinical Pharmacologist. Agencia de Ensayos Clínicos

Address: Planta 7ª Dcha Hospital Universitario Ramón y Cajal

Tel: 91 336 88 25

Fax: 91 336 88 25

[ipablo.hrc@salud.madrid.org](mailto:ipablo.hrc@salud.madrid.org)

**2. Title of the clinical trial**

# Pilot study of the effect of a CCR5 coreceptor antagonist on the latency and reservoir of HIV-1 in patients taking highly active antiretroviral therapy

**3. Protocol code**

ERRADVIH-01

EudraCT No. 2007-003995-21

**4. Chief investigator and address of center**

Santiago Moreno Guillén

Servicio de Enfermedades Infecciosas

Hospital Ramón y Cajal

Ctra. de Colmenar Km 9, 100

28034 Madrid.

Tel: +34 91 336 87 10

Fax: +34 91 336 87 92

[smoreno.hrc@salud.madrid.org](mailto:smoreno.hrc@salud.madrid.org)

**5. Centers where the study is to be carried out**

Servicio de Enfermedades Infecciosas, Hospital Ramón y Cajal.

**6. Institutional Review Board to evaluate the trial**

IRB of Hospital Hospital Ramón y Cajal.

**7. Name and credentials of the person responsible for monitoring**

Itziar de Pablo López de Abechuco.

Clinical Pharmacologist. Agencia de Ensayos Clínicos

Address: Planta 7ª Dcha, Hospital Universitario Ramón y Cajal

Tel: 91 336 88 25

Fax: 91 336 88 25

[ipablo.hrc@salud.madrid.org](mailto:ipablo.hrc@salud.madrid.org)

**8. Experimental drug and control drug**

Study drug:

Maraviroc (INN), 150 and 300-mg tablets, developed and supplied by Pfizer, S.A. One dose of 150-600 mg will be administered every 12 hours according to technical data and the previous antiretroviral treatment of each patient.

Control drug: Not applicable.

**9. Phase of trial**

Phase II clinical trial.

**10. Primary objective**

To evaluate the influence of treatment with a CCR5 coreceptor antagonist (Maraviroc) on the reservoir of HIV-1 in patients taking highly active antiretroviral therapy.

**11. Design**

Phase II Open Clinical Trial carried out in one centre

**12. Study pathology or disorder**

Infection by the human immunodeficiency virus (HIV)

**13. Primary endpoint**

Size of the latent reservoir measured as the number of resting CD4+ T lymphocytes containing a competent replicating virus.

**14. Study population and total number of patients**

Adult patients with HIV infection and a viral load that has been suppressed for a long period (less than 50 copies/mL for at least 2 years) while on antiretroviral therapy. The study will include 10 patients that maintain the habitual antiretroviral therapy combined with maraviroc.

**15. Duration of treatment**

12 months

**16. Calendar and expected end date**

After approval by the IRB and other regulatory bodies:

- Enrollment of patients: 6 months
- Duration of therapy and follow-up: 12 months

The study is expected to finish 18 months after approval.

# GENERAL INFORMATION

## **Identification of the trial**

**Protocol code:** ERRADVIH-01

**EudraCT no.:** 2007-003995-21

# Title: Pilot study of the effect of a CCR5 coreceptor antagonist on the latency and reservoir of HIV-1 in patients taking highly active antiretroviral therapy

## **Type of clinical trial**

Phase II open clinical trial carried out in one center

## **Study products**

**Study drug:**

Maraviroc (INN), 150 and 300-mg tablets, developed and supplied by Pfizer, S.A. One dose of 150-600 mg will be administered every 12 hours according to technical data and the previous antiretroviral treatment of each patient.

**Control drug:** Not applicable.

## **Sponsor data**

Fundación para la Investigación Biomédica del Hospital Ramón y Cajal

Address: Carretera de Colmenar Viejo Km 9, 100

28034 Madrid

**Contact**

Itziar de Pablo López de Abechuco.

Clinical Pharmacologist, Agencia de Ensayos Clínicos

Address: Planta 7ª Dcha, Hospital Universitario Ramón y Cajal

Tel: 91 336 88 25

Fax: 91 336 88 25

[ipablo.hrc@salud.madrid.org](mailto:ipablo.hrc@salud.madrid.org)

## **Technical director responsible for preparing and controlling the samples**

Pfizer Limited

Ramsgate Road,

Sandwich

CT13 9NJ

United Kingdom

## **Monitor**

Itziar de Pablo López de Abechuco.

María Angeles Gálvez Múgica

Farmacóloga Clínica, Agencia de Ensayos Clínicos

Address: Planta 7ª Dcha, Hospital Universitario Ramón y Cajal

Tel: 91 336 88 25

Fax: 91 336 88 25

[ipablo.hrc@salud.madrid.org](mailto:ipablo.hrc@salud.madrid.org)

## **Chief investigator and collaborators**

Chief Investigator:

Dr Santiago Moreno Guillén

Collaborating Investigators:

Dr José L. Casado Osorio

Dr Fernando Dronda Núñez

Dr Ana Moreno Zamora

Dr. M. Jesús Pérez Elías

Dra. Carolina Gutiérrez Montero

Dra. Beatriz Hernández Novoa

Dr. Rafael Rubio García

Dr. Alejandro Vallejo Tiller

Servicio de Enfermedades Infecciosas

Hospital Ramón y Cajal

Ctra. de Colmenar Km 9,100

28034 Madrid.

Tel: +34 91 336 87 10

Fax: +34 91 336 87 92

[smoreno.hrc@salud.madrid.org](mailto:smoreno.hrc@salud.madrid.org)

## **Study center**

Hospital Ramón y Cajal

## **Expected duration of the trial**

18 months

# RATIONALE AND OBJECTIVES

## **Origin of the project**

HIV-1 infection and AIDS are still among the most serious threats to mankind. To date, the AIDS pandemic has killed more than 28 million people and infected 42 million throughout the world. Despite prevention campaigns and new treatments, it is estimated that there will be 45 million new HIV-1 infections by the end of 2010.

Scientific advances in virology and molecular biology have made it possible to identify each step of the viral cycle and this has enabled us to develop combined drug therapy that acts on the different steps of the cycle (ie, reverse transcriptase and protease). This therapy, known as highly active antiretroviral therapy (HAART), has made it possible in approximately 70% of all treated patients to reduce the number of virus copies in plasma to levels that are undetectable using the most common molecular techniques (detection threshold: 50 copies of RNA/mL), thus reducing the morbidity and mortality associated with HIV infection and AIDS. Similarly, the development of increasingly potent antiretroviral drugs with more comfortable dosing schedules means that all antiretroviral treatment guidelines agree on recommending that the objective to be reached with HAART is an undetectable viral load in all patients, even in those whose treatment has already failed. Therefore, in developed countries, we now have drugs to maintain the level of viral replication below 50 copies of RNA/mL.

Patients who respond well to HAART enter a stable clinical phase with very low viremia and recovery of the immune system. Today, many HIV-1-infected patients remain clinically stable for several years with HAART. Nevertheless, this type of therapy does not act on the latent form of the virus, which is found in those cells that make up the so-called viral reservoir. Thus, when this therapy is interrupted in patients with an undetectable viral load, there is a rapid rebound in viremia and progression to AIDS, with the result that patients are obliged to maintain HAART indefinitely (Finzi et al., 1999). This treatment is very aggressive and has severe side effects that often lower the patient’s quality of life. Furthermore, it is very expensive.

Recently developed ultrasensitive techniques capable of detecting levels as low as 1 copy of RNA/mL have shown that although viral replication supposedly suppressed by HAART continues, it is still possible to detect viral replication below 50 copies. We do not know the origin of this replication, although there are 2 potential sources: release of viral particles from reservoirs where the virus was “latent” (reservoirs and sanctuaries), and residual replication that cannot be suppressed with available antiretroviral drugs (Siliciano JD, 2006). In theory, more potent antiretroviral therapy could be efficacious against residual replication, although it would continue to be inefficacious against a virus that is latently integrated in viral reservoirs.

Therefore, owing to the lack of effective vaccines, the development of new therapies that act on the viral reservoir is currently one of the most important challenges in the fight against AIDS.

**In vivo viral latency**

During primary infection, HIV disseminates to lymphoid and nonlymphoid tissue. It has been shown that, despite HAART in patients with no detectable viremia, HIV can be detected in cells of the CNS, genitourinary system, and lymphoid tissue associated with intestinal mucosa (Fierer DS, 2006; Galván SR, 2006; Blankson JN, 2006). One of the reasons why the virus is not eliminated from these sanctuaries could be the fact that antiretroviral drugs find it difficult to gain access (especially in the CNS) and also that infected cells escape the vigilance of the immune system. There is very little information on whether these reservoirs (sanctuaries) act as a source of residual replication in the presence of HAART or as pure reservoirs of latently integrated viruses.

From a clinical viewpoint, a small number of CD4^+^ memory cells are currently believed to represent the most important reservoir of HIV (Finzi et al., 1999). This reservoir has been calculated to be approximately 10^6^ cells, in other words, there is 1 cell reservoir for every 10^6^ resting CD4^+^ cells (Chun et al., 1997).

The therapeutic approach to viral latency requires viral reactivation in the presence of HAART (Yang QE, 2004), a selective attack with immunotoxins against latently infected cells (Brooks, et al., 2003), and intensification of HAART (Ramratnam et al., 2004), although this is far from being a reality in the treatment of AIDS.

Productive HIV infection in CD4^+^ cells is a complicated biological process that occurs when different cellular and viral events take place simultaneously. After the virus enters the host cell, the viral replication cycle will depend on cell activation status. Thus, infection of resting CD4^+^ cells gives way to incomplete viral reverse transcription, which represents a short period of viral latency with preintegrated HIV (Bukrinsky et al, 1991; Korin and Zack, 1998). If the cells are stimulated during the first 3 days after infection, the process of reverse transcription is completed and the viral cycle progresses to a productive infection (Namikawa et al., 1990). However, some of these infected cells return to a quiescent state (postintegration latency in memory cells) and represent one of the greatest problems for eradicating the virus from the body (Han et al., 2007).

This reservoir is established during the earlier phases of the infection and is obviously favored by the high viremia and hyperactivation of the immune system. The biology of the memory cells themselves means that the half-life of this reservoir has been calculated to be 44.2 months; therefore, the complete eradication of the reservoir would need 73.4 years of HAART (Siliciano et al., 2003). Nevertheless, these calculations depend on the real nature of the viral reservoir. In all patients studied with optimally performing HAART (the viral load in plasma is maintained below 50 copies of RNA/mL for several years), low levels of viremia may be due to residual replication, release of viruses from the reservoir, or a combination of both. Whereas some authors defend the idea that the reactivated viruses from the reservoir do not contribute to its subsequent maintenance (Siliciano, 2006) and, therefore, intensification of HAART would not be effective at reducing the size of the reservoir, other authors have shown that there is a clear communication between residual replication and maintenance of the reservoir, as exchange of viral particles between both compartments has been reported (Chun et al., 2005). According to these authors, the inclusion of integrase inhibitors and CCR5 coreceptor inhibitors would be expected to have a considerable influence on the dynamics and maintenance of this viral reservoir. This is one of the main objectives of this project.

**In vitro viral latency**

The main problem when attempting to understand the molecular mechanisms in viral latency is the lack of suitable models, as the frequency of latent infection in HIV-1-infected individuals is extremely low and neither the homogeneity of this reservoir nor the differences between individuals are well known. Nevertheless, groups including those of Jerome A. Zack and Eric Verdin have established 2 models that may indeed play an important role in the study of viral latency. The first is based on the SCID-hu murine model where liver and thymus tissue from human fetuses are implanted in SCID mice. These reconstitute an immune system that can be infected by HIV. In this model, some thymocytes contain inactive transcriptionally integrated virus that can replicate after cellular activation (Brooks et al., 2001). The second model uses a retroviral vector based on HIV to infect lymphoid cell lines by generating a cell clone library where expression of GFP correlates with the HIV-1 integration site (Jordan et al. 2003). This strategy has enabled us to establish a series of clones that express the virus latently and that can be reactivated using cell activation with TNFα or PMA (Jordan et al. 2003). These “latent” clones have been used in the study of the molecular mechanisms of viral latency (Williams et al., 2006) and for the study of the mechanisms of action of pharmacological compounds that act on viral latency (Williams et al., 2004, Márquez et al., 2007; Dauobi et al., 2007).

The use of lines latently infected with HIV has given way to new concepts about the molecular mechanisms that regulate viral latency. These concepts enrich our knowledge of this state of latency and are not completely exclusive—other models have been put forward. Thus, we have seen that p50, the subunit of NF-κB, interacts with HDAC1 and this complex favors maintenance of viral latency via changes in the structure of chromatin that prevent the recruitment of RNA pol II (Williams et al., 2006). It must be stressed that in primary resting lymphocytes there is an accumulation of p50 in the nucleus that acts by repressing genes that depend on NF-κB. When the cell is activated by the TCR or cytokines, the homodimers of p50 are substituted by heterodimers p50/p65 of NF-κB, thus producing the transcription of genes that depend on NF-κB, including HIV-1 LTR. In addition to p50, other factors such as YY1/LSF or even AP-4 can probably also recruit HDCA1 by favoring viral latency (He and Margolis, 2002; Imai and Okamoto, 2006). The absence of RNA pol II binding to latent LTR prevents the initiation of transcription, even in the presence of Tat (Williams et al., 2006). Therefore, viral latency may be governed by Tat levels and, especially, by the chromatin acetylation/deacetylation balance. This concept may explain the fact that trichostatin A (TSA) sensitizes latent LTR to the action of Tat. In other words, TSA favors the initiation of transcription required for the action of Tat on elongation (Williams et al., 2006).

It is worthy of note that these basic concepts can be extrapolated to the clinical management of viral latency. A recent study published by the RJ Bosch group has shown that valproic acid (VPA), an anti-epilepsy agent with HDAC-inhibiting action, can be effective for the treatment of viral latency in HIV-infected patients (Lehrman et al., 2005). This observation, although very limited by the number of patients involved, had been confirmed previously in vitro and therefore represents an important line of research in the treatment of viral latency. However, another, more recent study was not able to confirm the effect of VPA on the reduction in the size of the viral reservoir (Siliciano et al., 2007), Furthermore, the reduction in the reservoir observed by the Margolis group (Lehrman et al., 2005) was quite limited. Given that the reservoir must be almost 100% eradicated, it seems obvious that VPA alone could never be an option for eradicating the virus, although it could boost the effect of some pharmacological reactivators.

**Treatment of viral latency**

To date, clinical work on eradicating the viral reservoir has been based on the so-called immune activation therapy (IAT). This therapy has a double objective: first, it aims to induce viral replication and, thus, the new viruses produced would be sensitive to HAART; and second, the expression of viral antigens in the setting of MHC class-I would turn these cells into target cells of the immune response that would collaborate in the elimination of the viral reservoir. Several research groups have tried using the anti-CD3 monoclonal antibody OKT3 alone or in combination with IL-2, although without success. Another important candidate for the treatment of viral latency would be IL-7. This cytokine has been reported to be a potent reactivator of some HIV-1 subtypes in isolated T cells in patients taking HAART (Wang et al., 2005) and in the murine SCID-hu model of viral latency (Scripture-Adams et al., 2002). The latter group reported that combination therapy with IL-7 and immunotoxins induces depletion of the latently infected T cells (Brooks, et al., 2003).

An alternative to immunotherapy would involve drugs capable of reactivating viral latency, such as HDAC inhibitors (VPA) and some types of phorbol and diacylglycerol analogs (DAG), which do not present tumor-promoting activity. One of these derivatives is Prostratin (12-deoxyphorbol-13-acetate), which has been proposed for phase I clinical trials (AIDS Research Alliance, USA) (Hezareh, M. 2005). Prostratin induces reactivation of viral latency in vivo and in vitro (Brooks et al., 2001) by preferential activation of the PKCα and θ pathway and, as a consequence of this, the canonical activation pathway of transcription factor NF-κB is activated (Trushin et al., 2005). In addition to activating the NF-κB pathway, these deoxyphorbols induce endocytosis of HIV receptors (CD4 and CXCR4), which would mean that, as well as reactivation of the latent virus (susceptibility to HAART), reinfection of new CD4 cells would be avoided during the period of treatment with this type of compound (Hezareh, M. 2005). Despite the published data on the toxicity of prostratin and other deoxy-esters of phorbol in vivo, some authors maintain that, in order to reactivate viral latency, relatively high doses of this compound are necessary, which could be a serious limitation for development of the drug (Williams et al., 2004). Therefore, it would be interesting to study other more potent activators of viral latency, either alone or in combination with inhibitors of HDACs and/or IL-7, as proposed in some of the objectives of this study.

# Rationale

The presence of a pool of cells latently infected by HIV-1 in patients taking HAART and with a viral load below 50 copies/mL is the main limitation to eradication of the virus from the body. This viral reservoir prevents antiretroviral therapy from being interrupted; therefore, patients are obliged to continue with treatment for a period calculated to be greater than 60 years.

Despite the important advances in knowledge of the biology of this reservoir, we still have no real knowledge about its dynamics. The opportunity to carry out a clinical trial for the first time with CCR5 coreceptor antagonists is exceptional, since the results could provide important information on the nature of this reservoir.

If maintenance of the reservoir is a dynamic process, inclusion of CCR5 inhibitors is expected to lead to a reduction in the size of this reservoir. This effect could be critical when including IAT (viral reactivation), since, in theory, it would be necessary to act on a smaller reservoir. Current consensus is that it would be necessary to act on almost 100% of the viral reservoir (approximately 10^6^ cells).

The study has also been designed to enable us to understand the biochemical and molecular mechanisms by which certain drugs can induce viral reactivation in vitro as a previous step to a clinical trial aimed at reactivating viral latency and eradicating HIV-1 from the body.

**REFERENCES.**

1. Bukrinsky et al, 1991. Science, 254: 423.
2. Korin and Zack, 1998: J. Virol. 72: 3161.
3. Namikawa et al., 1990. J.Exp.Med. 172: 1055.
4. Finzi et al., 1999. Nat. Med. 5: 512.
5. Yang QE, 2004. Med Sci Monit; 10(7): RA155.
6. Ramratnam et al., 2004. J. Acquir Immune Defic Syndr 35: 33.
7. Brooks et al., 2001. Nat. Med. 7: 459.
8. Jordan et al. 2003. EMBO J 22: 1868.
9. Williams et al., 2006. EMBO J 25; 139.
10. He and Margolis, 2002. Moll Cell Biol. 22:2965.
11. Imai and Okamoto, 2006. J Biol Chem, 281: 12495.
12. Lehrman et al., 2005. Lancet 366: 549.

13. Scripture-Adams et al., 2002. J. Virol. 76; 13077.

14. Brooks, et al., 2003. Immunity, 19: 413.

# 15. Han Y,et al.,2007. Nat Rev Microbiol. 5(2):95.

# 16. Siliciano et al., 2003. Nat Med 9:727.

# 17. Chun et al., 1997. Nature 387; 183.

# 18. Chun et al., 2005. J. Clin. Invest. 115: 3250.

# 19. Márquez et al., 2007. Mol Pharmacol. In revision.

# 20. Daoubi et al., 2007. Bioorg. Med. Chem. In press.

21. Siliciano et al., 2007. J Infect Dis. 195(6):833.

22. Fierer DS and Klotman ME. 2006. Curr Opin HIV AIDS 1:115.

23. Galvin SR and Cohen MS. 2006. Curr Opin HIV AIDS 1:162.

24. Crowe SM. Curr Opin HIV AIDS 1:129.

## **Primary objective**

To evaluate, by means of a controlled clinical trial, the effect of therapy with a CCR5 coreceptor antagonist (Maraviroc) on the cell reservoir of HIV-1 on patients taking HAART.

## **Secondary objectives**

1. To quantify in vitro the viral reservoir of these patients throughout the study.

2. To study in vitro the residual viremia using an ultrasensitive PCR assay (Single Copy Assay) and the Episomal 2-LTRs DNA circles detection.

3. To study the immune activation markers as indirect markers of residual viremia

# TYPE OF TRIAL AND DESIGN

## **Clinical trial phase**

Phase II

## **Randomization process**

Not applicable

## **Type of control and design**

Not applicable

## **Blinding techniques**

Not applicable

## **Enrollment, washout, and follow-up**

The enrollment period will be 6 months. There will be no washout period. The initial follow-up will be 12 months.

# SELECTION OF SUBJECTS

## **Inclusion criteria**

- After receiving information on the design and objectives of the study, the possible risks involved, and the fact that they can refuse to collaborate at any time, patients will give their informed consent to participate in the study and agree to provide material for the cellular and molecular studies.
- Aged over 18 years.
- Chronic HIV infection
- Antiretroviral therapy with at least 3 drugs for at least 2 years and with no modifications expected during the study. Antiretroviral drugs can be switched due to intolerance as long as plasma viremia remains controlled.
- Undetectable viral load determined by ultrasensitive techniques (<50 copies HIV RNA/mL) for at least 2 years.
- CD4^+^ T lymphocyte count above 350 cells/mm^3^.
- Demonstration of R5 viral tropism (use of CCR5 coreceptors) or R4/dual tropism (use of CXCR4 or both coreceptors) by phenotyping in plasma samples stored before antiretroviral therapy is started.
- Understand the objective of the study and be available to make frequent visits to the hospital.

## **Exclusion criteria**

- Previous failure of antiretroviral therapy, understood as a rebound in viral load that can be detected after having reached undetectable levels. Low-grade increases (<200 copies of HIV RNA/mL) and transitory increases (blips) resolved without modifying antiretroviral therapy are excluded.
- Proven resistance against the antiretroviral drugs under study.
- Planned interruption of antiretroviral therapy.
- Taking immunosuppressive or immunostimulating medication of any type, including valproic acid.
- Taking a fusion inhibitor (enfuvirtide).
- Pregnancy or intention to become pregnant during the study.

## **Number of subjects planned and rationale**

As this is a pilot study, the minimum number of patients necessary to prove the concept has been estimated in 10.

## **Analysis and withdrawal criteria**

The patient can decide to withdraw from the study at any time. The investigator can also decide to withdraw a patient if the patient does not fulfill the norms of the protocol. Virological failure or development of toxicity to any of the study drugs will also be a reason for leaving the study.

## **Approximate duration of the enrollment period**

6 months.

# DESCRIPTION OF TREATMENT

## **Definition of treatment**

Maraviroc, tablets.

*Dose per subject*: 150 - 600 mg every 12 hours according technical data and previous treatment

*Route of administration*: Oral

## **Concomitant treatment**

The antiretroviral regimen that the patient had been taking and that must include at least 3 antiretroviral drugs (2 nucleoside reverse transcriptase inhibitors combined with a protease inhibitor or a non-nucleoside reverse transcriptase inhibitor).

# STUDY DEVELOPMENT AND EVALUATION OF RESPONSE

## **Evaluation criteria.**

The efficacy of the therapy administered will be evaluated by measuring the size of the latent reservoir of HIV-1. The frequency of resting CD4+ T cells infected by a replicative virus will be determined. The description of the samples and the technique to be used for their evaluation are included in Annex 4.

## **Study development**

Patients who fulfill the inclusion criteria will undergo the following procedures:

- Clinical history including duration of antiretroviral therapy and demonstrated period of undetectable viral load.
- Complete physical examination including height, weight, and temperature.
- Heart rate and systolic and diastolic blood pressure.
- Blood count, biochemistry, and urinalysis as described in section 8.3.2 of this protocol. Women will undergo a pregnancy test.
- Determination of viral load using ultrasensitive methods and CD4+ T lymphocyte count in blood samples.
- Determination of viral tropism.
- Measurement of the size of the reservoir of latent virus and its reactivation capacity.

Blood samples will be extracted by conventional phlebotomy on the dates set out in the study time line. The determinations will be carried out in the study center, with the exception of the determination of viral tropism and measurement of the size of the latent reservoir. For the former, samples will be sent to the Monogram® laboratories in the United States of America, as this is the only laboratory in the world approved for phenotyping coreceptors. For the latter, the samples will be taken in two laboratories: Infectious Diseases Laboratory from Hospital Ramón y Cajal and to the Molecular Immunobiology Laboratory of the Hospital General Universitario Gregorio Marañón (Dra. María Angeles Muñoz-Fernández) in Madrid.

After inclusion each patient receives, in addition to their usual medication, a CCR5 coreceptor antagonist entry inhibitor (maraviroc) in a dose of 150-600 mg every 12 hours according to technical data and previous treatment.

Treatment will be administered for at least 12 months. During this period, viral load will be measured using ultrasensitive methods, the CD4 count will be determined in peripheral blood, and the size of the reservoir of latent virus will be measured every 3 months after a blood extraction of 350 of total blood. After the intensification period with maraviroc and after drug suspension, patients will be following up during two additional visits according the study plan.

**STUDY PLAN**

| ***Day*** | ***Baseline***  ***1*** | ***Baseline 2*** | ***Month 3*** | ***Month 6*** | ***Month 9*** | ***Month 12*** | ***Month 15*** | ***Month 18*** |
| --- | --- | --- | --- | --- | --- | --- | --- | --- |
| **Informed consent** | X |  |  |  |  |  |  |  |
| **Clinical history** | X | X | X | X | X | X | X | X |
| **Physical examination^1^** | X | X | X | X | X | X | X | X |
| **Pregnancy test (women)** |  | X |  |  |  |  |  |  |
| **Determination of viral tropism** | X |  |  |  |  |  |  |  |
| **Inclusion criteria** | X |  |  |  |  |  |  |  |
| **Laboratory determinations^2^** | X | X | X | X | X | X | X | X |
| **Administration of drugs** |  | X | X | X | X | X |  |  |
| **Evaluation of adverse effects** |  | X | X | X | X | X |  |  |

^1^ Includes heart rate and blood pressure.

^2^ Includes blood count, biochemistry, habitual immunological and virological monitoring, as well as measurement of the size of the latent reservoir, residual viremia and immune activation markers.

## **Description of the methods for evaluating the response**

### Laboratory determinations

Blood workup: red cells, hemoglobin, hematocrit, mean corpuscular volume, mean corpuscular hemoglobin, mean corpuscular hemoglobin concentration, platelets, leukocytes, and leukocyte formula.

Biochemistry: aspartate aminotransferase, alanine aminotransferase, LDH, alkaline phosphatase, GGT, total bilirubin, uric acid, creatinine, urea, calcium, phosphorus, glycemia, cholesterol, triglycerides, and albumin.

Pregnancy test.

CD4^+^ T lymphocyte count.

Determination of HIV viral load using ultrasensitive methods.

Evaluation of viral tropism, by phenotyping.

Measurement of latent reservoir of HIV-1 (see Annex IV)

Direct measurement of residual viremia using the real time PCR (SCA) and Episomal DNA circles with 2-LTRs (see Annex IV)

Immune Activation Markers as indirect measurement of residual viremia (see Annex IV)

# These analyses will be carried out in the laboratories of Hospital Ramón y Cajal as part of the routine monitoring of antiretroviral therapy in HIV-infected patients. The only exceptions to this are the evaluation of viral tropism (Monogram, California USA) and measurement of the latent HIV reservoir, which will be carried out in an external laboratory (Molecular Immunobiology Laboratory, Hospital General Universitario Gregorio Marañón (Dra M. Angeles Muñoz-Fernández). The methods used to carry out these determinations are specified in Annex IV.

# ADVERSE EVENTS

## **Minimum required information**

The research team will follow up any adverse events that occur during the study. They will record the time of onset, the duration, intensity, course, and outcome of the event in order to evaluate the relationship of causality between the adverse event and the medication.

Recording of adverse events will be by a generic question of the following type: Have you noticed anything since you started taking the medication? or after spontaneous notification by the patient.

## **Imputability criteria**

Adverse events will be graded by their relationship of causality with the drug according to the algorithm of Karch and Lasagna (1977) as:

- *Definite*: There is a plausible time sequence between administration of the drug and onset of the adverse event—this event follows a known response pattern to the suspected drug, improves when the drug is suspended, and reappears when it is re-administered.
- *Probable*: There is a plausible time sequence between administration of the drug and onset of the adverse event—this event follows a known response pattern to the suspected drug, and cannot be explained otherwise.
- *Possible*: There is a plausible time sequence between administration of the drug and onset of the adverse event—this event follows a known response pattern to the suspected drug, but it can be explained otherwise.
- *Conditional or improbable*: There is a plausible time sequence between administration of the drug and onset of the adverse event—this event does not coincide with the adverse events described for the drug and can be explained otherwise.
- *Not related*: There is no plausible time sequence between administration of the drug and onset of the adverse event—this event does not coincide with the adverse reactions described for the drug and it can be explained otherwise.

The adverse event will also be classified according to its intensity:

*Mild*: Does not cause limitations to usual activity. Does not require additional treatment.

*Moderate*: Causes some limitation to daily activities and may or may not require further treatment.

*Severe:* Leads to inability to carry out daily activities and requires further therapy.

In general, **severe and/or life-threatening adverse reactions** will be considered to be those that are possibly or probably related to the medication and that fulfill any of the following criteria:

- an event that leads to death of the volunteer or patient
- an event that threatens the life of the volunteer or patient
- an event that requires hospitalization or prolongs existing hospitalization
- an event that causes permanent or significant disability or invalidity
- an accidental or voluntary overdose
- an event that reveals a congenital anomaly or birth malformation and malignant processes
- an important medical event, understood as an event that requires urgent intensive treatment

An **unexpected adverse event** is considered to be any experience not described (in its nature, severity, or frequency) in the product manual.

## **Procedures for the notification of adverse events**

Any suspicion of a severe and unexpected adverse event possibly related to the drug (adverse reaction) will be reported immediately by telephone to the study monitor. Within 3 days of the telephone report, a written, detailed, and suitably evaluated account of the event will be sent to the sponsor in accordance with RD 223/2004 and following the model set out in Appendix D published by the AEMPS (Spanish Medication Agency) to this effect (Appendix E of the protocol).

The investigator will be responsible for reporting the event to the monitor, sponsor, proprietary laboratory, the IRB of the study center, and the health authorities.

Initial reports and follow-up reports will identify the study subjects using a specific code for each of them.

The investigator will keep a detailed register of all adverse events. This register will be presented to the proprietary laboratory and the AEMPS on request.

## **Procedures for notification of severe and unexpected adverse events.**

The sponsor will notify the AEMPS of all suspected severe and unexpected adverse events associated with the study drugs in accordance with current legislation.

The maximum period for notification will be 15 days, except when the suspected severe and unexpected adverse event has lead to the death of the subject, or has placed the subject’s life in danger. In this case, the event will be reported within the 7 natural days after knowledge of the event. The information can be completed within the following 8 days.

Reports to the AEMPS will be by fax 91 822 50 76 or mail to Area de ensayos clínicos de la Subdirección General de Medicamentos de Uso Humano, Parque Empresarial Las Mercedes Edificio 8, C/Campezo 1 28022 Madrid.

Reports to the CAM will be via the contact person in Dirección General de Farmacia y Productos Sanitarios de la Consejería de Sanidad y Consumo de la CAM, Area de Investigación clínica y EPAs, Paseo de Recoletos, 14 - 2º, 28071 Madrid. Telephone: 91 426 9203 Fax: 91 426 9216 E-mail: [carmen.aguado@salud.madrid.org](mailto:carmen.aguado@salud.madrid.org).

Reports to the IRB of Hospital Ramón y Cajal: fax 91 336 88 25 / 91 336 83 22, at [ceic.hrc@salud.madrid.org](mailto:ceic.hrc@salud.madrid.org), or directly to Planta–2 Dcha IRB Secretary.

# ETHICS

## **General and specific norms for investigators**

The investigators will adhere strictly to the stipulations of this protocol, and will complete all the case report forms, which they will duly send to the sponsor or collaborating entity chosen by the sponsor to analyze the data.

The trial will be carried out in accordance with the recommendations for clinical trials and drug evaluation in humans, which appear in the Declaration of Helsinki (Appendix A), reviewed in Tokyo, Venice, Hong-Kong, South Africa, and Edinburgh (2000) and in current Spanish legislation on clinical trials.

## **Informed consent**

Before the study starts, all the participating subjects will be informed and will give their written informed consent. The informed consent form appears in Appendix D. The participant will receive a copy of this information sheet to take away.

## **Security and confidentiality**

The information published and obtained as a result of this study is considered confidential and must be treated as such at all times. The study subjects will be identified only by their study subject code. Both the investigators responsible for the clinical trial and a representative of the sponsor or the Health Authorities will have access to the information recorded during the study. If the results of the study are published, the identity of the volunteers will not be revealed.

## **Content of the trial budget**

Investigator remuneration for this trial etc. is specified in the financial report, Appendix B of the protocol.

## **Insurance**

The Fundación para la Investigación Biomédica of the Hospital Ramón y Cajal has a civil liability insurance policy fulfilling the requirements of Royal Decree 223/2004 that will provide compensation and indemnity to the participants in the case of damage to their health or any injuries they might suffer.

Furthermore, this policy covers the responsibilities of the sponsor, investigator team, and the Hospital Ramón y Cajal (Annex IV).

# PRACTICAL CONSIDERATIONS

## **Responsibilities of the trial participants**

The investigators will adhere to the norms of Good Clinical Practice and will know and follow the standardized operating procedures of the Infectious Diseases Service.

All the information recorded during the trial will be noted directly on the case report form (see Annex 1). When a correction is made, the person making the correction should write his/her initials and the date.

Auxiliary staff will follow the instructions given by the investigator concerning blood sample extractions, management thereof, and other complementary examinations.

Before the study, patients must receive oral and written information concerning the design and objectives of the study and the possible risks involved with it (Appendix D). If they then agree to participate, they must sign the consent form. This will not prevent them from later revoking this decision and leaving the study at any time and for any reason.

Patients will receive instructions on the need to strictly adhere to the instructions given by the investigators. They will be informed of the need to contact the investigators if any incidence arises during the study. The means to do this must be provided during the outpatient period of the study.

## **Deviations from the protocol**

The investigators present in these circumstances will make a complete report of the deviation and the reason for it in the CRF. If the deviation involves the inclusion/exclusion criteria, the investigators will contact the monitor by telephone to report on the deviation.

## **Filing of the documentation**

There is a document file for all the data. These will be on paper and computerized for 15 years after the study ends. This file must contain the following:

1. Ethics committee approval of the protocol and the informed consent form.
2. Copy of the approved written informed consent form and protocol with any applicable amendments.
3. Any study correspondence with the sponsor during the study.
4. Any correspondence with the ethics committee.
5. List of members of the ethics committee that approved the study protocol.
6. Signed acceptance of the protocol.
7. *Curriculum vitae* of the Chief Investigator and of the other investigators who make up the investigator team.
8. Register of the signatures of the members of the investigator team.
9. Reports of severe AE.
10. Contract between the sponsor and the investigator team.
11. List of the participant identities.
12. Copies of the CRF.

## **Identification of the samples**

The study medication will be provided by Laboratorios Pfizer, S.L., manufacturers of the product, and will be labeled according to the recommendations of Royal Decree 223/2004.

## **Amendments to the protocol**

Neither the investigator nor the monitor nor the sponsor will modify this protocol without the previous permission of the other parties. The modification should be recorded in writing. Any change in the research activity, except those necessary to eliminate an immediate apparent risk for the patient must be reviewed and approved by the ethics committee before their implementation. The sponsor must send the amendments to the protocol to the health authorities, and the modifications may need to be reviewed and approved by the ethics committee.

## **Acceptance by the investigator**

The investigator commitment is included in Appendix C.

## **Conditions for publication**

The results of the present study will be published, and the permission of the investigators and the sponsor will be required. A copy of the manuscript to be published must be made available to the monitor and the sponsor 15 days before it is sent to the editor. Similarly, the confidentiality of patient identity will always be respected.

# STATISTICAL ANALYSIS

This is an exploratory pilot study that is trying to prove a concept. Therefore, it is not possible to estimate the size of the sample necessary to demonstrate this. A treatment group of 10 patients has been considered necessary, based on data from previous studies on the same subject, which have used between 4 (Lehrman et al. Lancet 2005) and 11 (Chun et al. J Clin Invest. 2005) patients.

The longitudinal and cross-sectional analysis of the frequency data of infected cells will be carried out using random-effects regression models. In these models, the variable time will be introduced as an independent variable assuming linear kinetics. The frequencies of infected cells will be calculated using a parametric maximum likelihood method described elsewhere for limiting dilution experiments (Myers, L.E., McQuay, L.J. & Hollinger, F.B. Dilution assay statistics. J. Clin. Microb 1994; .32, 732–739) and will be expressed as infectious units per million (IUPM) of resting CD4 T cells. The gradient of the log_10_ reduction in IUPM will be estimated according to time from the start of therapy. This modelling process will be carried out in 2 approaches. In the first, 2 models will be made to estimate the gradients of log_10_ reduction in IUPM in the group of patients treated with the drug (n=10). Assuming linear reduction kinetics over time, the half-life will be calculated. The second approach will estimate a single model of the same characteristics as the previous ones but will include an interaction term between time and the treatment received. Given the design of the pilot study (concept), the statistical results will be reported as the estimations together with their standard errors without placing emphasis on the P values found. All the analyses will be carried out using STATA ver 9.0.

# APPENDICES

A. Declaration of Helsinki

WORLD MEDICAL ASSOCIATION DECLARATION OF HELSINKI

Ethical Principles for Medical Research Involving Human Subjects

Adopted by the 18th WMA General Assembly, Helsinki, Finland, June 1964, and amended by the
29th WMA General Assembly, Tokyo, Japan, October 1975
35th WMA General Assembly, Venice, Italy, October 1983
41st WMA General Assembly, Hong Kong, September 1989
48th WMA General Assembly, Somerset West, Republic of South Africa, October 1996
and the 52nd WMA General Assembly, Edinburgh, Scotland, October 2000
Note of Clarification on Paragraph 29 added by the WMA General Assembly, Washington 2002
Note of Clarification on Paragraph 30 added by the WMA General Assembly, Tokyo 2004

1. INTRODUCTION
   1. The World Medical Association has developed the Declaration of Helsinki as a statement of ethical principles to provide guidance to physicians and other participants in medical research involving human subjects. Medical research involving human subjects includes research on identifiable human material or identifiable data.
   2. It is the duty of the physician to promote and safeguard the health of the people. The physician's knowledge and conscience are dedicated to the fulfillment of this duty.
   3. The Declaration of Geneva of the World Medical Association binds the physician with the words, "The health of my patient will be my first consideration," and the International Code of Medical Ethics declares that, "A physician shall act only in the patient's interest when providing medical care which might have the effect of weakening the physical and mental condition of the patient."
   4. Medical progress is based on research which ultimately must rest in part on experimentation involving human subjects.
   5. In medical research on human subjects, considerations related to the well-being of the human subject should take precedence over the interests of science and society.
   6. The primary purpose of medical research involving human subjects is to improve prophylactic, diagnostic and therapeutic procedures and the understanding of the aetiology and pathogenesis of disease. Even the best proven prophylactic, diagnostic, and therapeutic methods must continuously be challenged through research for their effectiveness, efficiency, accessibility and quality.
   7. In current medical practice and in medical research, most prophylactic, diagnostic and therapeutic procedures involve risks and burdens.
   8. Medical research is subject to ethical standards that promote respect for all human beings and protect their health and rights. Some research populations are vulnerable and need special protection. The particular needs of the economically and medically disadvantaged must be recognized. Special attention is also required for those who cannot give or refuse consent for themselves, for those who may be subject to giving consent under duress, for those who will not benefit personally from the research and for those for whom the research is combined with care.
   9. Research Investigators should be aware of the ethical, legal and regulatory requirements for research on human subjects in their own countries as well as applicable international requirements. No national ethical, legal or regulatory requirement should be allowed to reduce or eliminate any of the protections for human subjects set forth in this Declaration.
2. BASIC PRINCIPLES FOR ALL MEDICAL RESEARCH
   1. It is the duty of the physician in medical research to protect the life, health, privacy, and dignity of the human subject.
   2. Medical research involving human subjects must conform to generally accepted scientific principles, be based on a thorough knowledge of the scientific literature, other relevant sources of information, and on adequate laboratory and, where appropriate, animal experimentation.
   3. Appropriate caution must be exercised in the conduct of research which may affect the environment, and the welfare of animals used for research must be respected.
   4. The design and performance of each experimental procedure involving human subjects should be clearly formulated in an experimental protocol. This protocol should be submitted for consideration, comment, guidance, and where appropriate, approval to a specially appointed ethical review committee, which must be independent of the investigator, the sponsor or any other kind of undue influence. This independent committee should be in conformity with the laws and regulations of the country in which the research experiment is performed. The committee has the right to monitor ongoing trials. The researcher has the obligation to provide monitoring information to the committee, especially any serious adverse events. The researcher should also submit to the committee, for review, information regarding funding, sponsors, institutional affiliations, other potential conflicts of interest and incentives for subjects.
   5. The research protocol should always contain a statement of the ethical considerations involved and should indicate that there is compliance with the principles enunciated in this Declaration.
   6. Medical research involving human subjects should be conducted only by scientifically qualified persons and under the supervision of a clinically competent medical person. The responsibility for the human subject must always rest with a medically qualified person and never rest on the subject of the research, even though the subject has given consent.
   7. Every medical research project involving human subjects should be preceded by careful assessment of predictable risks and burdens in comparison with foreseeable benefits to the subject or to others. This does not preclude the participation of healthy volunteers in medical research. The design of all studies should be publicly available.
   8. Physicians should abstain from engaging in research projects involving human subjects unless they are confident that the risks involved have been adequately assessed and can be satisfactorily managed. Physicians should cease any investigation if the risks are found to outweigh the potential benefits or if there is conclusive proof of positive and beneficial results.
   9. Medical research involving human subjects should only be conducted if the importance of the objective outweighs the inherent risks and burdens to the subject. This is especially important when the human subjects are healthy volunteers.
   10. Medical research is only justified if there is a reasonable likelihood that the populations in which the research is carried out stand to benefit from the results of the research.
   11. The subjects must be volunteers and informed participants in the research project.
   12. The right of research subjects to safeguard their integrity must always be respected. Every precaution should be taken to respect the privacy of the subject, the confidentiality of the patient's information and to minimize the impact of the study on the subject's physical and mental integrity and on the personality of the subject.
   13. In any research on human beings, each potential subject must be adequately informed of the aims, methods, sources of funding, any possible conflicts of interest, institutional affiliations of the researcher, the anticipated benefits and potential risks of the study and the discomfort it may entail. The subject should be informed of the right to abstain from participation in the study or to withdraw consent to participate at any time without reprisal. After ensuring that the subject has understood the information, the physician should then obtain the subject's freely-given informed consent, preferably in writing. If the consent cannot be obtained in writing, the non-written consent must be formally documented and witnessed.
   14. When obtaining informed consent for the research project the physician should be particularly cautious if the subject is in a dependent relationship with the physician or may consent under duress. In that case the informed consent should be obtained by a well-informed physician who is not engaged in the investigation and who is completely independent of this relationship.
   15. For a research subject who is legally incompetent, physically or mentally incapable of giving consent or is a legally incompetent minor, the investigator must obtain informed consent from the legally authorized representative in accordance with applicable law. These groups should not be included in research unless the research is necessary to promote the health of the population represented and this research cannot instead be performed on legally competent persons.
   16. When a subject deemed legally incompetent, such as a minor child, is able to give assent to decisions about participation in research, the investigator must obtain that assent in addition to the consent of the legally authorized representative.
   17. Research on individuals from whom it is not possible to obtain consent, including proxy or advance consent, should be done only if the physical/mental condition that prevents obtaining informed consent is a necessary characteristic of the research population. The specific reasons for involving research subjects with a condition that renders them unable to give informed consent should be stated in the experimental protocol for consideration and approval of the review committee. The protocol should state that consent to remain in the research should be obtained as soon as possible from the individual or a legally authorized surrogate.
   18. Both authors and publishers have ethical obligations. In publication of the results of research, the investigators are obliged to preserve the accuracy of the results. Negative as well as positive results should be published or otherwise publicly available. Sources of funding, institutional affiliations and any possible conflicts of interest should be declared in the publication. Reports of experimentation not in accordance with the principles laid down in this Declaration should not be accepted for publication.
3. ADDITIONAL PRINCIPLES FOR MEDICAL RESEARCH COMBINED WITH MEDICAL CARE
   1. The physician may combine medical research with medical care, only to the extent that the research is justified by its potential prophylactic, diagnostic or therapeutic value. When medical research is combined with medical care, additional standards apply to protect the patients who are research subjects.
   2. The benefits, risks, burdens and effectiveness of a new method should be tested against those of the best current prophylactic, diagnostic, and therapeutic methods. This does not exclude the use of placebo, or no treatment, in studies where no proven prophylactic, diagnostic or therapeutic method exists.
   3. At the conclusion of the study, every patient entered into the study should be assured of access to the best proven prophylactic, diagnostic and therapeutic methods identified by the study.
   4. The physician should fully inform the patient which aspects of the care are related to the research. The refusal of a patient to participate in a study must never interfere with the patient-physician relationship.
   5. In the treatment of a patient, where proven prophylactic, diagnostic and therapeutic methods do not exist or have been ineffective, the physician, with informed consent from the patient, must be free to use unproven or new prophylactic, diagnostic and therapeutic measures, if in the physician's judgement it offers hope of saving life, re-establishing health or alleviating suffering. Where possible, these measures should be made the object of research, designed to evaluate their safety and efficacy. In all cases, new information should be recorded and, where appropriate, published. The other relevant guidelines of this Declaration should be followed.

**B. Financial Report**

C. Commitment from Chief Investigator and Collaborators

D. Informed Consent Form

**PATIENT INFORMATION SHEET AND INFORMED CONSENT**

# Pilot study of the effect of a CCR5 coreceptor antagonist on latency and the reservoir of HIV-1 in patients taking highly active antirretroviral therapy

**SPONSOR:**

**FUNDACIÓN PARA LA INVESTIGACIÓN BIOMÉDICA DEL HOSPITAL RAMÓN Y CAJAL**

Protocol code: ERRADVIH-01

Versión 5.0 fecha: 31 enero de 2010

***INTRODUCTION***

We would like to inform you about the development of the clinical trial in which you are being asked to participate. Our intention is that you receive suitable and sufficient information so that you can evaluate and decide whether or not you wish to participate in this study. Therefore, please read this information form carefully and we will clarify any doubts that may arise after the explanation.

***BACKGROUND***

The clinical trial to be carried out is a study promoted by the Fundación para la Investigación Biomédica of Hospital Ramón y Cajal to determine if a new antiretroviral medication (used to treat HIV infection) can help to eliminate cells in which the virus is found in latent form (the so-called viral reservoir) and which, until now, has been the main barrier to eradicating HIV from the body.

This medication is called maraviroc and it belongs to a new family of antiretroviral drugs. Maraviroc prevents the virus from entering the cell. As HIV needs to enter cells to multiply, multiplication is severely affected in patients taking maraviroc. This drug is taken orally and has been tried on a large number of patients. Administration of the medication to patients with multiple treatment failures and who have resistance to most of the available drugs leads to a considerable fall in the quantity of virus in blood (viral load) and a marked increase in defenses (CD4+ lymphocytes). The medication is well tolerated, and in clinical trials it has not shown significant toxicity.

Apparently, this beneficial effect of maraviroc is similar to that obtained with other antiretroviral drugs that have been available for some time now. Therefore, why should this new medication have an additional effect on the viral reservoirs that other known medication does not have? This is due to its mechanism of action. Some researchers have shown that the viral reservoir is maintained and does not disappear, regardless of the treatment patients take, thanks to the continuous infection of new cells by the viruses remaining in the blood. If maraviroc blocks the entrance of viruses to new cells in the reservoir, the size of the reservoir might be considerably reduced over time. It seems likely that no drug alone would be capable of completely eliminating this viral reservoir and that several different medications may be necessary. However, it would be important to verify that the medications we use in habitual clinical practice could have such a marked effect.

This study is to be carried out only in Hospital Ramón y Cajal and will include 10 patients. All the patients will have an undetectable viral load and will continue with their habitual treatment. These patients are also going to take maraviroc, in addition to their habitual treatment.

You have being participated in the study, so now we ask you about a new informed consent to continue with two more determinations after 48 weeks intensification with the drug and the discontinuation.

***BENEFITS AND RISKS STEMMING FROM YOUR PARTICIPATION***

You are in good health (that is why you can participate in this clinical trial) and it is unlikely that this state of health will improve by your participation. As we said before, the most beneficial achievement of taking maraviroc is that, in the tests to be carried out, we may be able to show that your reservoir has decreased in size and that you have fewer viruses in your body. This may be important for the future, but it is not guaranteed.

As for adverse reactions, neither is it likely that you will notice any difference. As you have already been informed, maraviroc has proven to be very safe in clinical trials. In fact, in these studies it was compared with placebo (an imitation of the study drug that contains nothing) and was no more toxic than the placebo itself (in many cases the toxicity was even lower). The effect on the liver has been examined in particular and the drug has proven safe in this sense.

In clinical trials to date in which patients received the optimal basic therapy in addition to maraviroc, the adverse events detected were similar to those described with placebo: diarrhea (20%); nausea (20%); headache and fatigue (15%); and cough, fever, and dizziness (between 5% and 10%).

Furthermore, we inform you that the only diagnostic or therapeutic tests you will undergo will be blood extractions for the corresponding studies. You are expected to undergo extractions every 3 months, similar to what normally happens with your doctor at the clinic. The only difference is that, given all the studies to be carried out, we will need a larger amount of blood at each extraction (approximately 100 cc, about 10 tubes). No extra tests will be carried out.

During the study period (1 year), you will be asked on several occasions about the possible adverse effects that can appear. If necessary, during the study, you will receive medical attention in emergency room of the Hospital Ramón y Cajal.

***ALTERNATIVE TREATMENTS***

To date, there is no medication that has shown the effect we want to study in this clinical trial and that we can offer you. You will continue with your habitual therapy and if you do not participate in the trial, your habitual treatment will not be affected.

***DEVELOPMENT OF THE TRIAL***

In order to participate in the study you must fulfill all the inclusion criteria specified in the trial protocol. You are receiving written information, but you will also receive additional oral information and you will be able to clear up all your doubts on any aspect of the study. If you agree, you can sign the enclosed consent form.

The trial will last approximately 1 year and, therefore, there will be a total of 5 visits (1 at the beginning of the study and then 1 every 3 months). At each visit, you can tell us how you are tolerating the medication and whether you need to change, and we will take the blood samples. We will inform you about the progress of the study and, of course, about the progress of your viral load and defenses.

In January 2010 we had been proposed a relevant change in the study development. We ask you to a new written informed consent for two more laboratory determinations after 48 weeks intensification and discontinuation. The period determinations will be similar, each 3 months and with the same characteristics. This study modification will be useful to probe if the positive benefits of maraviroc intensification continue after the suspension.

***FINANCIAL COMPENSATION***

You will not receive financial compensation for your participation in the study. The study medication will be supplied to you free and there will be no extra visits due to the study.

***INSURANCE***

The Sponsor has an insurance policy that meets the requirements of current legislation (Royal Decree 223/2004) and will provide compensation and indemnity in the case of damage to your health or lesions that may occur.

***OTHER IMPORTANT CONSIDERATIONS***

If you decide to participate in this trial, you should know that you do so voluntarily and that you can **leave at any time**. You should also know that you can be excluded from the trial if the study investigators feel that it is appropriate, whether for reasons of safety, because of any adverse advent resulting from the study medication, or because they consider that you are not adhering to the established procedures, since, when you signed the enclosed consent form, you **made a commitment to fulfill the study procedures set out therein.**

All the data you provide us with, both in the preparation of your clinical history and for the rest of the study documents will be filed in our Service, on paper and on computer. Data collected for the study will be identified using a code and only the chief investigator/collaborators will be able to connect these data with you and with your clinical history.

The data will be included in a database that complies with Law 15/1999 on Protection of Personal Data. Similarly, data will be transmitted with the appropriate security measures in conformance with this law and with RD 994/99. Only those data from the clinical history related to the study can be checked. This check will be made to every possible extent in the presence of the Chief Investigator/Collaborating Investigators, who will be responsible for guaranteeing the confidentiality of all the data from all the clinical histories belonging to the subjects participating in the trial. You will have the right to access, rectify, and cancel your data at any time.

All samples will be processed in the Laboratory from the Infectious Diseases Department. The only people who can have access to your data are members of the Infectious Diseases Service, who manage the database and who are obliged to maintain the confidentiality of the information contained therein. A representative of the Fundación para la Investigación Biomédica del Hospital Ramón y Cajal and/or the Health Authorities can have access to the trial data by means of a numeric code to maintain anonymity. If the study results are published, your identity will not be released.

If you have any doubts about your rights, please contact the Institutional Review Board or the Patient Service Department of the Hospital.

**Women must undertake to use contraception or to abstain from sexual relations in order not to become pregnant**. The contraceptive method will be a barrier method (condom), which, in addition, avoids transmission of HIV.

You will be informed of any new information relevant to the study drugs that is discovered during the course of the study.

***CONTACTS***

At all times, you will have access to a team of health care professionals who you must not hesitate to ask to clarify any doubts you may have about the medication, food and drink you can take, etc. If you need any information or for whatever other reason, do not hesitate to contact the chief investigators of the study Dr. Moreno in the telephone 91 336 87 11, or in the mobile 661 51 52 24.

**Patient’s signature**: **Investigator’s signature**:

**Name**: **Name**:

**Date**: **Date**:

**This document will be signed in duplicate. One copy will be given to the investigator and the other to the patient**

***WRITTEN INFORMED CONSENT MODEL***

# Title of the Trial: Pilot study of the effect of a CCR5 coreceptor antagonist on the latency and reservoir of HIV-1 in patients taking highly active antiretroviral therapy.

Protocol code: ERRADVIH-01

**Sponsor:** Fundación para la Investigación Biomédica del Hospital Ramón y Cajal

I (name and surnames)

..........................................................................................................................

have read the information sheet that I have been given.

have had the opportunity to ask questions about the study.

have received sufficient information about the study.

have spoken with

............................................................................................

(name of the investigator)

I understand that my participation is voluntary.

I understand that I can withdraw from the study

a) whenever I want,

b) without having to give explanations,

c) with no repercussions for my medical care,

I freely agree to participate in the study.

DATE : PARTICIPANT’S SIGNATURE

DATE : INVESTIGATOR’S SIGNATURE

Appendix E. **Annex D Form for the notification of a severe and unexpected adverse event in Spain**

| NOTIFICATION OF SUSPECTED ADVERSE REACTION FOR **STUDY MEDICATION** | PROTOCOL CODE (sponsor) | NOTIFICATION NO. (Sponsor) |
| --- | --- | --- |
|  | PATIENT NO. | NOTIFICATION NO. |

1. INFORMATION ON THE ADVERSE REACTION

| 1a. COUNTRY | 2. DATE OF BIRTH | | | 2a. AGE | 3. SEX | 3a. WEIGHT | 3b. HEIGHT | 4-6. DATE REACTION STARTED | | | |
| --- | --- | --- | --- | --- | --- | --- | --- | --- | --- | --- | --- |
|  | DAY | MONTH | YEAR |  | 🞎 MALE  🞎 FEMALE |  |  | DAY | | MONTH | YEAR |
| 7. DESCRIPTION OF THE ADVERSE REACTION (Including relevant results from the examination or from the laboratory, and the end date, if applicable). | | | | | | | | | 8-13b. CRITERIA OF SEVERITY/OUTCOME  🞎 DEATH  🞎 PATIENT’S LIFE HAS BEEN IN DANGER  🞎 HOSPITALIZATION  🞎 PROLONGATION OF HOSPITALIZATION  🞎 PERMANENT OR SIGNIFICANT DISABILITY  🞎 CLINICALLY RELEVANT ADVERSE REACTION  🞎 PERSISTENCE OF THE ADVERSE REACTION  🞎 RECOVERY | | |

**II. INFORMATION ON THE STUDY MEDICATION**

| 14. MEDICATION SUSPECTED OF CAUSING ADVERSE REACTION | 15. DAILY DOSE | 16. ROUTE | 17. STUDY PATHOLOGY | | 18. DATES  START END | | 19. DURATION OF THERAPY |
| --- | --- | --- | --- | --- | --- | --- | --- |
|  |  |  |  | |  |  |  |
| 20. DID THE REACTION DISAPPEAR WHEN THE MEDICATION WAS SUSPENDED?  🞎 YES 🞎 NO 🞎 NOT APPLICABLE | | 20a. DID THE REACTION DISAPPEAR WHEN THE DOSE WAS REDUCED?  🞎 YES 🞎 NO 🞎 NOT APPLICABLE | | 21. DID THE REACTION REAPPEAR WHEN THE MEDICATION WAS RE-ADMINISTERED?  🞎 YES 🞎 NO 🞎 NOT APPLICABLE | | | |

**III. CONCOMITANT MEDICATION AND CLINICAL HISTORY**

| 22. CONCOMITANT MEDICATION (Mark with an asterisk the medication (s) suspected of causing the adverse reaction) | 22a. DAILY DOSE | 22b. ROUTE | 22c. DATE  START END | | 22d. REASON FOR THE PRESCRIPTION |
| --- | --- | --- | --- | --- | --- |
|  |  |  |  |  |  |
|  |  |  |  |  |  |
|  |  |  |  |  |  |
|  |  |  |  |  |  |
| 23. IMPORTANT DATA FOR THE CLINICAL HISTORY (eg, diagnoses, allergies, pregnancies, etc.) | | | | | |

**IV. INFORMATION ON THE SPONSOR AND INVESTIGATOR**

| 24a. NAME AND ADDRESS OF THE SPONSOR | | 24b. NAME AND ADDRESS OF THE INVESTIGATOR |
| --- | --- | --- |
| 24c. LABORATORY CODE  (AEM NO.) | 25a. TYPE OF REPORT  INITIAL  FOLLOW-UP | 24c. SPONSOR’S REPORTING TECHNICIAN  NAME:  TELEPHONE:  SIGNATURE: |
| 24e. DATA OF REPORT | 24f. DATE OF ENTRY TO AEM | 25b. COMPLEMENTARY REPORT ENCLOSED |

**GENERAL INSTRUCTIONS**

1. This form will only be used to report suspected severe and unexpected adverse reactions that occur with the study medications.

2. Any suspicion of fatal or life-threatening reactions (those that would have led to the patient’s death if there had no been an immediate therapeutic intervention) will be reported within a maximum of 7 natural days; if all the information is not available, it can be completed within a further 8 days. Any other suspected severe and unexpected adverse events will be reported within a maximum period of 15 days.

3. When the there is not enough space, an additional information sheet will be added. This will be correctly identified with the name of the sponsor and the number assigned to the notification. This additional information can contain the evaluation of causality made by the reporting technician.

**SPECIFIC INSTRUCTIONS**

1. The protocol code is that assigned by the sponsor to identify the trial. The sponsor notification number is the one the sponsor uses for filing. In the case of follow-up information, the same number will be used or, if this is modified, the number of the initial notification will be indicated. The shaded space “Notification No.” will remain empty.

2. Age will be in years, months, weeks, or days, as appropriate, but it will always be shown. If the exact age is not known, then at least the age group should be given (eg, infant, child, adolescent, adult, elderly person).

7. The adverse reaction will be reported fully, with its end date and the results of complementary examinations or laboratory tests that may be of interest. This notification can be accompanied by as many reports as considered necessary for a suitable interpretation of the symptoms suspected of being an adverse reaction.

8-13. The categories are not mutually exclusive. Attendance at a hospital emergency department for less than 24 hours is not considered hospitalization.

14. The study medications will be identified, if possible, by their generic name (DOE [Spanish nonproprietary name] or INN) indicating the commercial name when available or, if this is not possible, the product’s proposed name or laboratory code.

15. If administration of the drug is not daily, then it should be described with one of the following possibilities: cyclical, weekly, annual, or number of times used (in the latter case, the dose at each administration must be recorded, not the total).

17. The medical condition of the patient who is to receive the study drug must be stated. If there is none, then “healthy volunteer” must be used.

1. The duration of treatment until the onset of the adverse reaction must be stated.

22. If concomitant drugs have not been taken, then this must be stated explicitly. If any of the concomitant drugs are considered suspicious, they will be marked with an asterisk (eg, * AMOXICILLIN). The medications used to treat the adverse reaction will be excluded.

**ANNEX IV**

**REPORT ON THE ANALYSIS OF SAMPLES AND PROCEDURES TO BE USED**

**HIV-1 LATENT RESERVOIR QUANTIFICATION**

Despite the clinical importance of the latent reservoir of HIV-1 in resting CD4 T cells, no simple method for measuring the level of latently infected cells is available to the clinician. By definition, latently infected cells are in a state of reversible non-productive infection and therefore do not release virions. In the case of HIV-1, latently infected cells produce little HIV-1 mRNA and the mRNA that is produced seems to be prematurely terminated or mislocalized. Therefore, latently infected cells probably do not produce viral proteins and are indistinguishable form uninfected cells. This feature, together with their low frequency, greatly complicates their detection and quantification.

**Detecting latently infected cells in vivo**

To prove that latently infected cells are present, it is generally necessary three methodological steps:

1- A pure populations of resting CD4 T cells must be isolated

2- The presence of cells with integrated HIV-1 DNA must be demonstrated

3- It must be shown that replication-competent virus can be rescued from the resting CD4 T cells

**1. Isolated and purification of CD4 T cells**

CD4 T cells that are isolated from peripheral blood or lymphoid tissues of infected individuals contain a complex mixture of cells in various states if differentiation, activation and infection, so for that reason the detection and quantification of latently infected cells is greatly facilitated by the purification of resting CD4 T cells.

Given that different activation markers appear and disappear from the cell surface with different kinetics, it is important to use markers that detect various stages of activation.

In this regard, CD69, CD25 and HLA-DR are used as markers of early, intimidated and late stages of T-cell-activation, respectively. To increase the purity of isolation, flow citometric separation is particularly useful because it allows the simultaneous gating of cells of a small, uniform size, other characteristics that distinguishes resting from activated CD4 T cells.

When resting CD4 T cells are isolated from patients on HAART who have clinically undetectable levels of viraemia, the resulting populations will not produce virus and in fact express little functional virus RNA. Therefore, replication competent virus rescued from such cells by activating stimuli can be said to have come latently infected cells.

The situation is more complex when resting cells are isolated from viraemic patients. Resting cell population from viraemic patients can produce low levels of viremia.

Latency can be most easily demonstrated in populations of resting CD4 T cells from patients on suppressive HAART regimens because these cells do not produce virus without stimulation.

**2. Detection of integrated HIV-1 DNA**

The second step in demonstrating the presence of latently infected cells within a given populations of infected cells is to detect integrated HIV-1 DNA in some cells in the population.

Standard PCR assays for HIV-1 DNA do not distinguish between integrated and unintegrated forms, and in viraemic patients it is likely that most of the HIV-1 DNA detected by standard PCR is in the unintegrated form.

Several assays have been developed to selectively detect integrated HIV-1 DNA. One of them is the inverse PCR assay in which the genomic DNA is digested with a restriction enzyme that leaves a 4-base overhang. The DNA is then diluted so that an intramolecular ligation can take place. Amplification with specific primers targeting HIV-1 gives rise to products that contain the junction between HIV-1 and host DNA. Another approach that has been widely used to demonstrate the presence of integrated HIV-1 DNA is *Alu*-PCR. This involves amplification using a primer in the common *Alu* retroelement and a primer in HIV-1. Both methods permit to distinguish between integrated and unintegrated HIV-1 DNA, but with the problem of both of them generate amplicons of different sizes according to the different integration sizes.

However, until now, there is no well validated and universally accepted method to quantify the number of cells carrying integrated HIV-1 DNA.

**3. Detection of cells carrying replication-competent virus.**

The most crucial aspect of measuring HIV-1 latency is demonstrated that once the infected resting CD4 T cells are activated, they can release replication competent virus. This is particularly important because much of the HIV-1 DNA in resting CD4 T cells is thought to be defective or inactivated. It is therefore important to demonstrate that replication-competent virus can be rescued from resting CD4 T cells by cellular activation.

Enhanced culture assay was used to quantify the frequency of cells harbouring replication competent virus, which included a step to induce the uniform activation of resting CD4 T cells, allowing rescue of latent virus.

Uniform activation can be achieved by the addition of the mitogen phytohematoglutinin (PHA) and irradiated allogenic peripheral blood mononuclear cells (PBMCs) from healthy donors. Following activation, PBMCs from healthy donors are added to the culture periodically to amplify any virus that is released following the activation of latently infected cells. Virus growth is detected by assay of an HIV-1 antigen (p24) in the culture supernatant after 2-3 weeks.

Globally the assay is a set up in a limiting dilution format so that frequencies can be measured, and therefore the culture conditions must be such that virus from a single latently infected cell can be efficiently expanded.

This assay is useful to study the latent reservoir in patients under suppressive HAART regimens because there are few recently infected resting CD4 T cells that contain unintegrated HIV-1 DNA. So in this situation virus that is rescued from resting CD4 T cells by cellular activation is probably derived from cells carrying replication competent viral genome. However in viraemic patients, the infected cells that are detected in the assay include both cells comprising the stable latent reservoir and recently infected cells containing labile, unintegrated HIV-1 DNA. Consequently, the frequencies of latently infected cells detected in viraemic patients are much higher than the frequencies of latently infected cells detected in patients on suppressive HAART regimens.

**STUDY METHODS**

**LATENTLY HIV-1-INFECTED MEMORY T CELLS QUANTIFICATION**

Two baseline determinations (three months apart) will be performed to investigate the accuracy of the method and the stability of the reservoir. We carried out a previously described co-culture assay. Briefly, PBMC will be isolate from 300 ml of heparinized whole blood using Ficoll density gradient centrifugation (Lymphocytes Isolation Solution, Rafer, S.L Zaragoza, Spain). Resting CD4^+^ T cells (CD4^+^/HLA-DR^-^/CD25^-^) will be isolate and purified using magnetic beads according to the manufacturer’s recommendations (Miltenyi Biotec, S.L. Bergisch Gladbach, Germany). CD4^+^ T-cell purity greater than 99% with less than 0.4% activation will be confirmed by flow cytometry. The isolated resting CD4^+^ T cells will be plate in replicate limiting dilutions and co-culture with allogenic irradiated PBMC from seronegative donors and phytohemaglutinin (PHA). The co-culture will be incubating with 5% of CO_2_ and 37ºC.

The activated cell culture will be fed once a week with PBMC from healthy donors after depletion of CD8^+^ T cells. On days 15 and 21, culture supernatants will tested for HIV-1 replication using the HIV-1 p24 antigen assay (Innogenetics Diagnostica Iberia, S.L Tarragona, Barcelona, Spain). Infected cell frequencies will be determine by the maximum likelihood method and expressed as infectious units per million (IUPM) resting CD4^+^ T cells. The accuracy of the measurement will depend on the number of replicates tested at each cell dilution.

RESIDUAL VIREMIA QUANTIFICATION

# RT-PCR real time ultrasensitive (“Single copy assay”)

# Residual viremia was measured with an ultrasensitive quantitative real-time RT-PCR (single copy assay), as reported previously by Dr Sarah Palmer et al (J Clin Microbiol 2003; 41: 4531-6). The optimal HIV-1 RNA detection threshold was 0.3 copies/ml when 5 to 7 ml of starting plasma was available. So we will use this volume from each patient and with a ultracentrifugation step sample concentration. Retrotranscription and amplification will be performed in two steps, so we amplified the study region using a real time quantitative PCR using the primers and fluorescent labels previously described by the authors. The lineal range will be from 1 to 10^6^ copies RNA HIV-1/ml.

**HIV-1 EPISOMAL 2LTR DNA CIRCLES DETECTION**

The presence of HIV-1 episomal 2LTR DNA circles will be detected by qualitative PCR. Enriched 2LTR episomal DNA circles will be extracted selectively from PBMC using QIAprep Spin Miniprep (Qiagen, Valencia, California, USA) following the manufacturer’s protocol for low-copy-number plasmids, as previously described Sharkey et al. [reference 12 ].

A nested PCR flanking the episomal 2LTR DNA circle junction will design. In the first round, 5–20 μL of episomal DNA will amplified in a 50-μL reaction with the following primers: forward, 5’-TAAGATGGGTGGCAAGTGGTCA; and reverse, 5’-TCTACTTGTCCATGCATGGCTT.

The second PCR round was performed using 1 to 2 μL of the first reaction product as the template and primers spanning the unique junction formed by ligation of 5’ and 3’ LTR sequences (forward, 5´-AATCTCTAGCAGTACTGGAAG; reverse, 5´-GCGCTTCAGCAAGCCGAGTCCT). PCR products were analyzed on a 1% agarose gel stained with GelRed (Biotium, Hayward, California, USA).


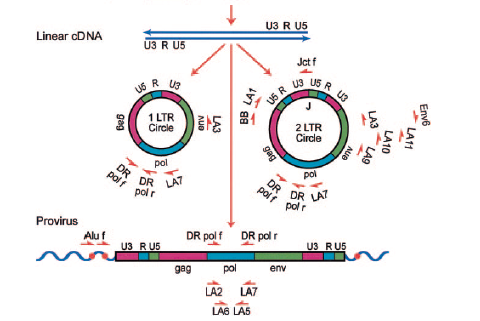


**IMMUNE ACTIVATION AND LYMPHOCYTE SUBSETS ANALYSIS**

Fresh EDTA anticoagulated whole blood will be used to analyze CD4^+^ and CD8^+^ T cell activation with the following antibody combination: CD3-allophycocyanin (APC)-Cy7, CD4-peridinin chlorophyll protein complex (PerCP), CD8-phycoerythrin (PE)-Cy7, CD38-phycoerythrin (PE), and HLA-DR-allophycocyanin (APC). Lymphocyte subpopulations will be defined as naïve (CD45RA+CCR7+), T central memory (CD45RA-CCR7+, TCM), T effector memory (CD45RA-CCR7-, TEM), and T effector memory RA+ (CD45RA+CCR7-, TemRA). To analyze lymphocyte subsets this antibody combination we will used: CD3-allophycocyanin (APC)-Cy7, CD4-peridinin chlorophyll protein complex (PerCP), CD8-phycoerythrin (PE)-Cy7, CD45RA- phycoerythrin (PE), and CCR7-allophycocyanin (APC), β7-allophycocyanin (APC). Antibodies will be from Becton Dickinson (Becton Dickinson, NJ, USA), and an unstained control will be performed for all samples. Briefly, 100 µL of blood were lysed with 200 µl of FACS Lysing solution (Becton Dickinson) for 30 min at room temperature, will be incubate with the antibodies during 20 min at 4ºC, wash and resuspend in PBS containing 1% azida. Cells will be analyzed in a Gallios flow cytometer (Beckman-Coulter, CA, USA). At least 15000 CD3^+^ cells were collected for each sample and analyzed with Kaluza software (Beckman-Coulter) initially gating lymphocytes according to morphological parameters.
